# Enhancing agency and empowerment in agricultural development projects: A synthesis of mixed methods impact evaluations from the Gender, Agriculture, and Assets Project, Phase 2 (GAAP2)

**DOI:** 10.1016/j.jrurstud.2024.103295

**Published:** 2024-05

**Authors:** Agnes R. Quisumbing, Ruth Meinzen-Dick, Hazel J. Malapit, Greg Seymour, Jessica Heckert, Cheryl Doss, Nancy Johnson, Deborah Rubin, Giang Thai, Gayathri Ramani, Emily Myers, GAAP2 for pro-WEAI Study Team, Agnes Quisumbing, Ruth Meinzen-Dick, Hazel Malapit, Malick Dione, Jessica Heckert, Hazel Malapit, Elena M. Martinez, Audrey Pereira, Greg Seymour, Chloe van Biljon, Ana Vaz, Marlène Elias, Ruth Meinzen-Dick, Annet Mulema, Emily Myers, Deborah Rubin, Ara Go, Federica Argento, Akhter Ahmed, Anika Hannan, Shalini Roy, Masuma Younus, Alan de Brauw, Amita Dey, Berber Kramer, Mike Murphy, Benjamin Crookston, Megan Gash, Bobbi Gray, Marwan Benali, Pepijn Schreinemachers, Caroline Sobgui, Sarah Janzen, Neena Joshi, Nicholas Magnan, Rajendra Pradhan, Sudhindra Sharma, Sophie Theis, Marc Bellemare, Bart Casier, Susan James, Brooke Krause, Mathias Lardinois, Aine McCarthy, Sabine Gabrysch, Sheela Sinharoy, Jillian Waid, Amanda Wendt, Josué Awonon, Rasmane Ganaba, Aulo Gelli, Elena Martinez, Abdoulaye Pedehombga, Armande Sanou, Sita Zougouri, Silvia Alonso, Alessandra Galiè, Tasokwa Kakota, Jef Leroy, Giordano Palloni, Elizabeth Bryan, Dawit Mekonnen, Mamun Miah, Neha Kumar, Saiqa Siraj, Mihret Alemu, Brenda Boonabaana, Ana Paula de la Ocampo, Susan Kaaria, Marya Hillesland, Erdgin Mane, Vanya Slavchevska, Avijit Choudhury, Madhu Khetan, Neha Kumar, Kalyani Raghunathan, Giang Thai

**Affiliations:** a International Food Policy Research Institute, Washington DC, USA; b Tufts University, Medford MA, USA; c Independent consultant, Minneapolis, MN USA; d Cultural Practice, LLC, Bethesda, MA, USA; e University of Minnesota, Minneapolis, MN, USA

## Abstract

Development interventions increasingly include women’s empowerment and gender equality among their objectives, but evaluating their impact has been stymied by the lack of measures that are comparable across interventions. This paper synthesizes the findings of 11 mixed-methods impact evaluations of agricultural development projects from South Asia and sub-Saharan Africa that were part of the Gender, Agriculture, and Assets Project, Phase 2 (GAAP2). As part of GAAP2, qualitative and quantitative data were used to develop and validate the multidimensional project-level Women’s Empowerment in Agriculture Index (pro-WEAI), which was used to assess the impact of GAAP2 projects on women’s empowerment. This paper assesses the extent to which: (1) a two- to three-year agricultural development project can contribute to women’s empowerment; and (2) a suite of methods comprising a standardized quantitative measure of women’s empowerment and a set of qualitative protocols, can evaluate such impacts. Our synthesis finds that the most common positive significant impacts were on the instrumental and collective agency indicators that comprise pro-WEAI, owing to the group-based approaches used. We found few projects significantly improved intrinsic agency, even among those with explicitly stated objectives to change gender norms. Unsurprisingly, we find mixed, and mostly null impacts on aggregate pro-WEAI, with positive impacts more likely in the South Asian, rather than African, cases. Our results highlight the need for projects to design their strategies specifically for empowerment, rather than assume that projects aiming to reach and benefit women automatically empower them. Our study also shows the value of a suite of methods containing a common metric to compare empowerment impacts and qualitative protocols to understand and contextualize these impacts.

## Highlights

• We use pro-WEAI to assess 11 projects' impacts on women's agency and empowerment.
• Pro-WEAI comprises quantitative and qualitative protocols co-developed by projects.
• Significant positive impacts observed on instrumental and collective agency.
• More limited or null impacts detected on intrinsic agency.
• Intentionality is important in projects' strategies to empower women.

## 1 Introduction

Many agricultural development interventions aim to empower women alongside goals to improve agricultural productivity and income; reduce poverty, hunger, and undernutrition; and improve health outcomes (Malapit et al., 2019). Initially considered a radical concept, women's empowerment has gained wider acceptance, first as instrumental to achieving more tangible outcomes, and more recently as intrinsically valuable, consistent with the Sustainable Development Goals (SDGs) (Cornwall, 2016; Cornwall and Edwards, 2014). Donors and international organizations have included “empowering women” among their program objectives, even if development projects themselves do not directly empower women (Cornwall 2016) but provide them opportunities to empower themselves.

To know whether agricultural development projects can meet their empowerment objectives, it is important to evaluate whether women participating in these projects indeed become more empowered within the typical time frame of project implementation. Efforts to assess whether agricultural development projects can empower women, and what is needed for this to happen, require syntheses across impact evaluations. An impact evaluation of a single project cannot provide a definitive answer. However, these efforts at synthesis have been stymied by several factors. First, most projects do not clearly define what they mean by empowerment (Malhotra et al., 2002) and often do not distinguish between reaching, benefitting, and empowering women (Johnson et al., 2018). Projects that reach women include them in program activities; those that benefit them improve women's well-being, such as income, health, and nutrition. But neither “reaching” nor “benefiting” women explicitly empowers them by increasing women's agency, their ability to make strategic life choices (Kabeer, 1999) and act on them. Second, although there are many approaches to evaluating empowerment impacts (see, for example, Alsop and Heinsohn, 2012; Elias et al., 2021; Hillenbrand et al., 2015; Holland and Ruedin, 2012; Ibrahim and Alkire, 2007; Lombardini et al., 2017; Mosedale, 2005, 2014; Narayan, 2005), until recently, no internationally validated measure of women's empowerment existed that was suitable for impact evaluations focusing on individual empowerment outcomes. Moreover, although empowerment is a multidimensional concept, most development funders and multilateral organizations emphasize economic empowerment (Bayissa et al., 2018; UNDP, 2011; Narayan, 2005). Third, most studies that collect individual-level data collect it only on women, so they cannot assess impacts on women's outcomes relative to men's. Fourth, with few exceptions, most impact evaluations are conducted on single projects using disparate empowerment measures, making it difficult to generalize or compare findings across projects. The exceptions are Lombardini and McCollum (2018), who use their version of a Women's Empowerment Index to evaluate empowerment impacts across Oxfam projects, and Quisumbing et al. (2023), who evaluate impacts across four projects in the UN's Joint Program for Rural Women's Economic Empowerment (JP RWEE) using the abbreviated Women's Empowerment in Agriculture Index (A-WEAI). Systematic reviews attempt to synthesize findings using meta-analysis and other techniques, but very few portfolios of projects with similar women's empowerment objectives are evaluated using a comparable and unified framework or use standardized or validated measures of women's empowerment across the entire portfolio. Finally, attempts to use a comparable measure across contexts has been hindered by the culture- and context-specificity of empowerment (Laszlo and Grantham, 2017) and discontent with quantitative measures that do not reflect local meanings of empowerment (O'Hara and Clement, 2018).

This paper synthesizes the findings of mixed-methods impact evaluations from the Gender, Agriculture, and Assets Project, Phase 2 (GAAP2), a multi-year (2015–2022) and multi-country project that aimed to improve projects' ability to meet and evaluate progress on their women's empowerment and gender equality objectives. Under GAAP2, researchers and project implementors collaboratively developed and validated the project-level Women's Empowerment in Agriculture Index (pro-WEAI) (Doss and Rubin, 2022; Malapit et al., 2019; Meinzen-Dick et al., 2019; Yount et al., 2019) using qualitative and quantitative data. This process led not only to the development of the quantitative index, pro-WEAI, but also accompanying, and considerably more flexible, qualitative protocols. The pro-WEAI suite of methods includes both the quantitative index and the qualitative protocols and we use it to assess the empowerment impacts of a portfolio of development projects with explicit objectives and strategies around women's empowerment (Johnson et al., 2018; Meinzen-Dick et al., 2019).

This paper addresses the following questions: First, to what extent can a two-to three-year agricultural development project contribute to women's empowerment? Second, to what extent can a suite of methods comprising a standardized quantitative measure of women's empowerment and a set of flexible qualitative protocols evaluate such impacts? More specifically, what insights can be gained from qualitative research to understand whether and how agricultural development projects can attain their empowerment objectives? What do estimated impacts reveal about what empowerment strategies have been proven effective across a variety of contexts?

We first present the Reach, Benefit, Empower, and Transform (RBET) framework, the types of strategies that projects in the GAAP2 portfolio used to attain their empowerment objectives, and the specific interventions implemented by projects. We discuss the pro-WEAI and the aspects of agency that it captures. We discuss the impacts of 11 GAAP2 projects on the three types of agency that pro-WEAI measures—intrinsic, collective, and instrumental—and on aggregate measures of women's empowerment and gender parity. Reflecting our mixed-methods approach, we draw from and triangulate across findings from the quantitative studies that estimated impacts on the pro-WEAI indicators^2^ as well as the qualitative studies conducted by each project. Where the findings differ, we do not try to establish which method is “right,” but rather engage with the differences to reveal a more complex picture of the empowerment process. We examine what the results mean both for the ability of projects to contribute to women's empowerment and for pro-WEAI as a measure of project impact. By considering both the quantitative and qualitative findings, we assess whether the quantitative impact measures captured men's and women's perceptions of project impact and aim to uncover any unanticipated impacts or pathways to empowerment. We conclude by reflecting on the lessons learned from using mixed methods to evaluate empowerment impacts across a project portfolio using a co-developed metric of empowerment, the pro-WEAI, together with its associated qualitative protocols.

## 2 The Gender, Agriculture, and Assets Project, Phase 2 (GAAP2) portfolio

This section describes the GAAP2 portfolio and the impact evaluation methods used by projects within the portfolio. Details on the portfolio and specifics on the impact evaluation methods used by each project are in Appendix Tables 1 and 2, respectively.

### 2.1 Project portfolio, strategies, and activities

To develop and validate pro-WEAI, GAAP2 worked with a portfolio of thirteen agricultural development projects that had explicit women's empowerment objectives. The projects identified their needs for qualitative and quantitative tools that would help them to understand their impacts on women's empowerment and sought to learn what works best, in terms of measurement and implementation, under different conditions. The projects were implemented in nine countries in South Asia and Africa, with multiple focal outcomes and different start and end dates. All projects aimed to improve women's empowerment and nutrition outcomes; some also sought to improve incomes. When projects applied to join GAAP2, project implementers and their evaluation partners identified the strategies that their projects used to empower women. The first stage of the qualitative analysis involved examining project documents to identify the gender-related strategies. This review led to the development of the “Reach-Benefit-Empower” framework used to classify strategies and activities (Johnson et al., 2018); this was subsequently expanded to include “Transform” objectives (Morgan et al., 2023; Quisumbing et al., 2023).

In this framework (Table 1), projects that *reach* women include them in program activities; those that *benefit* them improve women's well-being outcomes, including income, health, and nutrition. Typical indicators for “reach” include the number of women and men attending training or extension programs; “benefit” indicators include income earned by women or women's nutritional status indicators. But neither “reach” nor “benefit” objectives explicitly address empowerment, and many projects that claim to *empower* women only have strategies to reach or benefit them. Projects that aimed to empower women would need to go beyond “reach” and “benefit” and facilitate women being able to make and implement strategic life choices. Finally, gender-transformative approaches comprise a new category that was not in the original RBE framework. These approaches emphasize interventions that aim to *transform* formal and informal systems, institutions, and markets (Morgan et al., 2023). This could involve transforming gender norms, attitudes, and behaviors that limit women's opportunities towards those that support gender equality (10.13039/501100015815CGIAR Research Program on Fish Agri-food systems, 2017, quoted in Pyburn and van Eerdewijk, 2021). These approaches typically adopt a holistic approach to change gender norms at the community and societal levels, address structural and institutional barriers, and mobilize the power of the collective. Most projects conceptualize “empower” as occurring at the individual level, albeit in the context of a community, whereas transformation occurs at a higher level and involves changes in norms and structures and may lie beyond the purview of individual agricultural development projects. Thus, even if one could argue that transforming gender norms is a prerequisite to empowerment, this is typically beyond the scope of what individual agricultural development projects can achieve, unless they are implemented at scale.

**Table 1:** The reach, benefit, empower, and transform (RBET) framework.

Reach	Reach	Benefit	Empower	Transform
Definition	Include women in program activities	Increase women's wellbeing	Strengthen ability of women to make life choices and put them into action	Go beyond the woman and her household to change systems, gender norms and power relations on a larger scale
Objective	Ensures that women have the same opportunity to access the program activities as men: • Address barriers to participation, e.g. program information, timing or location of meetings and training	Requires more than reaching women: • Women value the intervention • Direct benefits accrue to women • Women's needs, preferences and constraints are considered in the intervention design	Goes beyond reaching and benefiting women: • Increases women's agency • Shifts gender norms and attitudes among participants	Goes beyond empowering individual women: • Involves men • Changes gender norms at community and societal levels • Addresses structural and institutional barriers • Mobilizes the power of the collective

We further classified project strategies into categories of influencing gender norms, building capacity (knowledge and skills), providing goods and services, and strengthening organizations (Table 2). We then identified which aspects of agency—intrinsic, instrumental, or collective—were targeted by that strategy.^3^ We classified project strategies to help implementers and evaluators think more carefully about their theories of change regarding women's empowerment and thereby enhance learning and, ultimately, project effectiveness. All projects completed qualitative studies prior to the COVID-19 pandemic, but two projects were unable to complete their endline surveys in time for the synthesis work on this project. This synthesis is therefore based on the 11 projects that completed endline data collection before December 2020.^4^

**Table 2:** Activity areas and specific activities according to RBET framework and type of agency targeted, GAAP2 portfolio.^a^.

Activity area	Specific activity/Link to RBET framework	Type of agency targeted	Number of projects using activity as part of their strategy (out of 13)^b^
Influence gender norms (G)	Awareness raising about gender issues and their implications (E, possibly T)	Intrinsic	3
	Community conversations to identify community solutions to gender issues (E, possibly T)	Intrinsic, possibly collective	8
Build capacity, knowledge, and skills (C)	Agricultural training and extension (R, possibly B)	Instrumental	10
	Business and finance training (R, possibly B)	Instrumental	6
	Nutrition education (R, possibly B)	Instrumental	8
	Other training (R, possibly B)	Instrumental	4
Provide goods and services (P)	Direct provision of goods/assets to beneficiaries (B, possibly E)	Instrumental	7
	Direct provision of services to beneficiaries (B)	Instrumental	5
	Indirect provision by supporting availability, quality, or access (R, B)	Instrumental	2
Strengthen organizations (S)	Form/strengthen groups or other organizations (such as enterprises) (R, possibly B, E)	Collective, possibly intrinsic	8
	Form/strengthen platforms or networks that link organizations (R, possibly B, E)	Collective	1

a B=Benefit, E = Empower, R=Reach, T = Transform.

b The strategy analysis includes all 13 projects in the GAAP2 portfolio. Projects may adopt multiple strategies.

The 11 completed GAAP2 projects were implemented in South Asia (Bangladesh (three), India, Nepal), West Africa (Burkina Faso (two), Ghana, Mali), and East Africa (Ethiopia, Tanzania). All but one of the partner projects worked through nongovernmental organizations (NGOs); most of them used group-based approaches, though not exclusively with women's groups. The lone partner project implemented by the government was the ANGeL project in Bangladesh, albeit with an NGO partner in one of the treatment arms. Details on the projects' interventions, mapped to type of strategy and type of agency targeted, are in Table 3.

**Table 3:** Treatment arms and project strategies in the GAAP2 portfolio, projects that completed impact evaluations.^a^.

Project acronym	Project objective	Approach	Type of strategy by treatment arm and estimation method^b^^,^^c^	Type of agency targeted
**South Asia/Bangladesh**				
ANGeL(Quisumbing et al., 2021)	To pilot alternative approaches to integrating agriculture, nutrition, and women's empowerment, with view to scaling up most effective approach	Provide training using three different approaches (treatments); all trainings delivered to husbands and wives jointly	Agriculture training (C; R, B)	Instrumental
			Nutrition BCC (C; R, B)	Instrumental
			Agriculture-Nutrition BCC (C; R, B)	Instrumental
			Agriculture-Nutrition BCC-Gender sensitization (C, G; R, B, E, T)	Instrumental, intrinsic
AVC (de Brauw et al., 2019)	To increase agricultural output and income, and improve food and nutrition security through strengthened agricultural value chains	Conduct trainings to build farmers' capacity in using improved seed varieties and cultivation practices, basic training on gender and nutrition issues, and provision of promotional discounts on fertilizer and seeds to incentivize adoption	Value chain promotion only	None
			NGO training only (G; E)	Intrinsic
			Training + promotion (G; E)	Intrinsic
FAARM (Wendt et al., 2019; Waid et al., 2022)	To reduce undernutrition among women and young children through a food-based dietary diversification strategy and to increase the status of women within the household	Train rural women's groups in vegetable gardening, fruit tree production, and poultry rearing, along with nutrition and hygiene	Homestead food production (C, G, S, P; R, B, E, T)	Intrinsic, instrumental, collective
**South Asia/India**				
WINGS (Kumar et al., 2021)	To improve women's and children's diets and nutrition outcomes by increasing own consumption and income Existing SHG platform has women's empowerment objectives	Using existing women's self-help groups, deliver BCC and training on nutrition-sensitive agricultural planning; work with the community and public systems/institutions to ensure that services of public health and nutrition programs are available and accessible in the project area	Nutrition intensification (DID) (C, G, S, P; R, B, E)	Intrinsic, instrumental, collective
			Nutrition intensification (SD) (C, G, S, P; R, B, E)	Intrinsic, instrumental, collective
**South Asia/Nepal**				
Heifer (Janzen et al., 2018a, 2018b)	To increase income, food security and nutrition, and women's empowerment, and improve aspirations, hope, and economic resilience among the chronically poor by building physical, human, and social capital	Provide women with livestock transfers and training related to nutrition, home gardening, and livestock management; form self-help groups through which women receive empowerment training	Full treatment-Direct beneficiaries (C, G, S, P; R, B, E)	Intrinsic, instrumental, collective
			Full treatment-Pay it forward beneficiaries (C, S, P: R, B)	Instrumental, collective
			Goats-Direct beneficiaries (C, S, P; R, B)	Instrumental, collective
			Goats-Pay it forward beneficiaries (C, S, P; R, B)	Instrumental
			Values-based training-Direct beneficiaries (C, G, S; R, E)	Intrinsic, instrumental, collective
			Values-based training-Pay it forward beneficiaries (S; E)	Instrumental, collective
**West Africa/Burkina Faso**				
Grameen (Crookston et al., 2021)	To increase the resilience of vulnerable communities in disaster-affected regions by building women's economic empowerment, and to strengthen women's capacity to make decisions about children's nutrition	Use community-based women's savings groups as a platform for improving livelihoods through training, education on agriculture as a business, linkages to agricultural services, financing for common agricultural activities, nutrition education, and gender dialogues	Treatment: women's savings groups, education, financing, nutrition education, gender dialogues (C, G, S, P; R, B, E)	Intrinsic, instrumental, collective
SELEVER (Gelli et al., 2017; Heckert et al., 2023)	To increase poultry production and improve the nutritional status of women and children in the Centre-Ouest, Hauts-Bassins and Boucle de Mouhoun regions of Burkina Faso	Use an integrated market-facilitation approach combining revenue generation, women's empowerment, and nutritional behavior change interventions	SELEVER (C, G, S; R, B, E)	Intrinsic, instrumental, collective
			SELEVER + (includes WASH) (C, G, S; R, B, E)	Intrinsic, instrumental, collective
**West Africa/Ghana**				
iDE (Bryan and Mekonnen 2022)	To expand production of food during the lean season and reduce production risks during rainy seasons through small-scale irrigation, to increase income, food security, nutrition, and health	Provide women access to motor pumps along with training, access to credit, and other agricultural inputs	Motor pump - control group 1 – DID (S, P; R, B)	Instrumental, collective
			Motor pump - control group 1 – SD (S, P; R, B)	Instrumental, collective
			Motor pump - control group 2 – DID (S, P; R, B) (S, P; R, B)	Instrumental, collective
			Motor pump - control group 2 – SD (S, P; R, B)	Instrumental, collective
			Motor pump - spillover – DID (S, P; R, B)	Instrumental, collective
			Motor pump - spillover – SD (S, P; R, B)	Instrumental, collective
**West Africa/Mali**				
WorldVeg (Benali et al., 2020)	To improve nutritional status and dietary diversity by increasing vegetable production and consumption	Integrated home garden project—combining training in gardening with nutrition behavior change communication and training in water, sanitation, and hygiene (WASH)	Home garden project with training in nutrition BCC and WASH-ITT (C, G, S, P; R, B, E)	Intrinsic, instrumental, collective
			Home garden project with training in nutrition BCC and WASH-ToT (C, G, S, P; R, B, E)	Intrinsic, instrumental, collective
**East Africa/Ethiopia**				
JP RWEE (Hillesland et al., 2022)	To reduce gender inequalities in pastoralist communities related to access to resources, credit, and financial services to improve household food security, women's decision making within the household, and women's participation in the community	Strengthen associations and cooperatives to offer financial products to women farmers, provide credit to women farmers, and give women financial literacy and entrepreneurship training	Beneficiaries who lost access to credit (C, G, S, P; R, B, E, T)	Intrinsic, instrumental, collective
			Beneficiaries with access to credit (C, G, S, P; R, B, E, T)	Intrinsic, instrumental, collective
			Married women/men - Beneficiaries who lost access to credit (C, G, S, P; R, B, E, T)	Intrinsic, instrumental, collective
			Married women/men - Beneficiaries with access to credit (C, G, S, P; R, B, E, T)	Intrinsic, instrumental, collective
**East Africa/Tanzania**				
Maisha Bora (Krause et al., 2018, 2020)	To increase food security of semi pastoralist communities through a more diversified and secure income from improvements in livestock	Build capacity of pastoralists' organizations to provide entrepreneurship training, business skills training, and advocacy for women; form savings and credit groups and women-only farms; provide training on household budgeting and gender awareness	Intervention (strengthen organizations, provide training, form credit groups, gender training) (C, G, S, P: R, B, E)	Intrinsic, instrumental, collective

a Only the 11 projects that completed their endline surveys by December 2020 are included in this table.

b Aims abbreviations: B=Benefit, E = Empower, R=Reach, T = Transform. Strategy abbreviations: C=Build capacity, knowledge and skills; G = Influence gender norms, P=Provides goods and services, S=Strengthens organizations.

c Where projects used different estimation procedures as robustness checks, these are indicated in separate rows. DID = Difference in Difference; SD= Single difference; ITT = intent to treat; ToT = Treatment effect on the treated.

### 2.2 Project evaluation design across the GAAP2 portfolio

GAAP2 worked with each project's existing evaluation design, adding top-up funding to implement pro-10.13039/100006006WEAI quantitative and qualitative protocols (Meinzen-Dick et al., 2019). Appendix Table 2 presents the evaluation designs used by the 11 completed projects, as well as the qualitative protocols that they used. The evaluation designs fall into two main categories: (1) randomized controlled trials (six projects), and (2) quasi-experimental difference-in-difference designs (five projects, of which three used matching methods and/or inverse probability weights, one used entropy balancing, and one did not use any matching or weighting procedure). In all cases, the control group was clearly established so that empowerment impacts could be assessed relative to a well-defined counterfactual. Treatment arms used in the impact evaluations are listed in Table 4.

**Table 4:** Strategies employed by projects by type of agency.

Project	Treatment arm/Estimation method	Type of agency and project strategy				
		Intrinsic	Instrumental		Collective	
		Influence gender norms	Build capacity	Provide goods and services	Strengthen organizations	
ANGeL - A	Agricultural extension	No	Yes	No	No	
ANGeL - N	Nutrition BCC	No	Yes	No	No	
ANGeL - AN	Agriculture and nutrition training	Yes	Yes	No	No	
ANGeL - ANG	Agriculture, nutrition, and gender sensitization training	No	Yes	No	No	
AVC - Training	NGO trainings only	No	No	No	No	
AVC - Promotions	NAAFCO promotions only	Yes	No	No	No	
AVC - T + P	Trainings + promotions	Yes	No	No	No	
FAARM	Homestead food production program	Yes	Yes	Yes	Yes	
WINGS - DD	Nutrition-intensification - Double difference	Yes	Yes	Yes	Yes	
WINGS - SD	Nutrition-intensification - Single difference	Yes	Yes	Yes	Yes	
Heifer - DFull	Full treatment - Direct beneficiaries	Yes	Yes	Yes	Yes	
Heifer - DTrain	Values based training - Direct beneficiaries	No	Yes	Yes	Yes	
Heifer - DGoat	Goats - Direct beneficiaries	No	Yes	Yes	Yes	
Heifer - PIFFull	Full treatment – Pay-it-forward beneficiaries	No	Yes	Yes	Yes	
Heifer -PIFTrain	Values based training – Pay-it-forward beneficiaries	Yes	Yes	No	Yes	
Heifer - PIFGoat	Goats – Pay-it-forward beneficiaries	No	No	No	Yes	
Grameen	Intervention	Yes	Yes	Yes	Yes	
SELEVER	SELEVER	Yes	Yes	No	Yes	
SELEVER+	SELEVER+	Yes	Yes	No	Yes	
iDE - DD1	Motor pump, relative to control group 1 - DiD	No	No	Yes	Yes	
iDE - DD2	Motor pump, relative to control group 2 - DiD	No	No	Yes	Yes	
iDE - DDS	Motor pump, relative to spillover - DiD	No	No	Yes	Yes	
iDE - SD1	Motor pump, relative to control group 1 - SD	No	No	Yes	Yes	
iDE - SD2	Motor pump, relative to control group 2 - SD	No	No	Yes	Yes	
iDE - SDS	Motor pump, relative to spillover - SD	No	No	Yes	Yes	
WorldVeg - ITT	Intervention, intent-to-treat estimate (ITT)	Yes	Yes	Yes	Yes	
WorldVeg - ToT	Intervention, treatment effect on the treated estimate (ToT)	Yes	Yes	Yes	Yes	
JP-RWEE – All, credit	All women/men - Beneficiaries with access to credit	Yes	Yes	Yes	Yes	
JP-RWEE – All, lost credit	All women/men - Beneficiaries who lost access to credit	Yes	Yes	Yes	Yes	
JP-RWEE - Married, credit	Married women/men - Beneficiaries with access to credit	Yes	Yes	Yes	Yes	
JP-RWEE – Married, lost credit	Married women/men - Beneficiaries who lost access to credit	Yes	Yes	Yes	Yes	
Maisha Bora	Intervention	Yes	Yes	Yes	Yes	
Number of projects with strategy		10	9	8	9	
(% of 11)		(90.9)	(81.8)	(72.7)	(81.8)	
No of treatment arms with strategy		18	22	21	25	
(% of 32)		(56.3)	(68.8)	(65.6)	(78.1)	
No. of treatment arms with at least one strategy		31				
(% of 32)		(96.9)				
No. of treatment arms with any two strategies		7				
(% of 32)		(21.9)				
No. of treatment arms with any three strategies		6				
(% of 32)		(18.8)				
No. of treatment arms with all four strategies		12				
(% of 32)		(37.5)				

The main quantitative metric of women's and men's empowerment is pro-WEAI (Malapit et al., 2019). At the GAAP2 inception workshop in 2016, participating projects critiqued the existing Women's Empowerment in Agriculture Index (WEAI) questionnaire and proposed additional domains and indicators that they deemed essential to project success. Program implementers and quantitative and qualitative researchers who have studied women's empowerment collaboratively designed a new survey instrument by proposing content to pilot. The project teams field-tested the new materials in their project baselines, conducted between April 2016 and June 2018. Sample sizes for the baseline surveys that implemented pro-WEAI ranged from 380 women and 380 men in the Grameen Foundation project to 1487 women and 1396 men in SELEVER, both implemented in Burkina Faso. The projects followed guidelines for a minimum sample size of 350 households to achieve a sample size that was large enough for index construction and validation (Malapit et al., 2017), following the protocol used to develop the original WEAI ((Alkire et al., 2013).

Projects also implemented qualitative protocols (Appendix Table 3) as part of developing the pro-WEAI suite of methods. To develop these protocols, the research team held a virtual meeting in April 2016, to discuss lessons learned from previous qualitative work and objectives for future qualitative research. These protocols ended up being implemented at different stages in each project's lifecycle and with different priority research topics.^5^ For those projects that were able to field the pro-WEAI survey module at baseline, the qualitative work helped ensure that pro-WEAI reflected the aspects of empowerment participants deemed most important. The qualitative protocols were designed to be flexible to accommodate different project contexts, the sequencing and timing of quantitative and qualitative methods, and any additional objectives of the qualitative research.

Baseline quantitative data were then shared with the pro-WEAI team for analysis, validation, and creation of draft pro-WEAI index and component indicators. Feedback on the draft index was elicited from the participant projects and expert stakeholders in the research and development communities.^6^ Some projects that began earlier used A-WEAI in their baselines and fielded pro-WEAI at endline. Because A-WEAI can be computed from the same data used for pro-WEAI, difference-in-difference estimates can be used to assess impact, although some projects (e.g. ANGeL) took advantage of the randomized design to estimate single-difference impacts using pro-WEAI at endline.^7^ Except for Heifer, which ended earlier, all projects implemented pro-WEAI at endline.

Qualitative work was an important part of the impact evaluation protocols; where qualitative work was not originally part of the projects' impact evaluation design, GAAP2 provided top-up funding to conduct it. All projects conducted key informant and/or life history interviews, as well as focus group discussions. Across all projects, 453 interviews and 166 focus group discussions were conducted (Appendix Table 4). FAARM conducted more interviews than initially planned, to attain saturation^8^ on preliminary emerging themes that were not initially anticipated. Although time and budget constraints, as well as the availability of certain categories of respondents, limited the ability of the researchers to fully reach saturation, most projects found sufficient repetition among interviewees' and focus group participants’ responses, suggesting all significant themes were covered. The pro-WEAI qualitative protocols are not prescriptive in terms of how the analysis should be conducted. Our recommendation was that analysis should be led by a trained qualitative researcher who is an expert in the local context, but in cases where such a person could not be identified, someone with qualitative analysis expertise worked with field teams with local knowledge. Transcripts and field notes were analyzed for common themes and reviewed for patterns that emerged.

Most projects integrated qualitative work while the project was underway, as part of a process evaluation or to interpret project impacts. The qualitative findings shed light not only on the impacts of the project on specific aspects of agency, but also the interrelationships among various aspects of agency. Although qualitative work undertaken as part of pro-WEAI did not inform the design of the interventions, the implementation partners had been working in the study sites for a long time and knew the contexts of women's lives.

The quantitative and qualitative findings did not always agree. The divergences stimulated further consideration by the research teams, based on the assumption that each method offered a different but equally valid perspective. The analyses in each case reported the diverse findings and sought to account for the differences. An example from the Nepal project illustrates this process well: qualitative data clearly indicated that daughters-in-law were disempowered, especially in time use, but the quantitative data did not show significant differences in empowerment overall or in the workload indicator. Further investigation showed that quantifying workloads in terms of hours spent in productive and domestic work did not capture the (lack of) control over time, ultimately suggesting further work on measuring time agency (Doss et al. 2020).

## 3 Measuring agency and empowerment using pro-WEAI

The pro-WEAI (Malapit et al., 2019) is rooted in Kabeer's (1999) definition of empowerment and its three dimensions of resources, agency, and achievements and focuses specifically on three domains of agency: intrinsic, instrumental, and collective. These domains correspond to Rowlands' (1995) classification of generative types of power, which includes “power within” (enhancing self-respect, self-efficacy, and an awareness of rights), “power to” (enacting personal goals and creating new opportunities), and “power with” (acting collectively toward shared goals) (see also, Ibrahim and Alkire, 2007). Pro-WEAI focuses on measures of agency because metrics for resources and achievements are well developed. In this paper, we use “agency” and “empowerment” interchangeably.

**Fig. 1:**
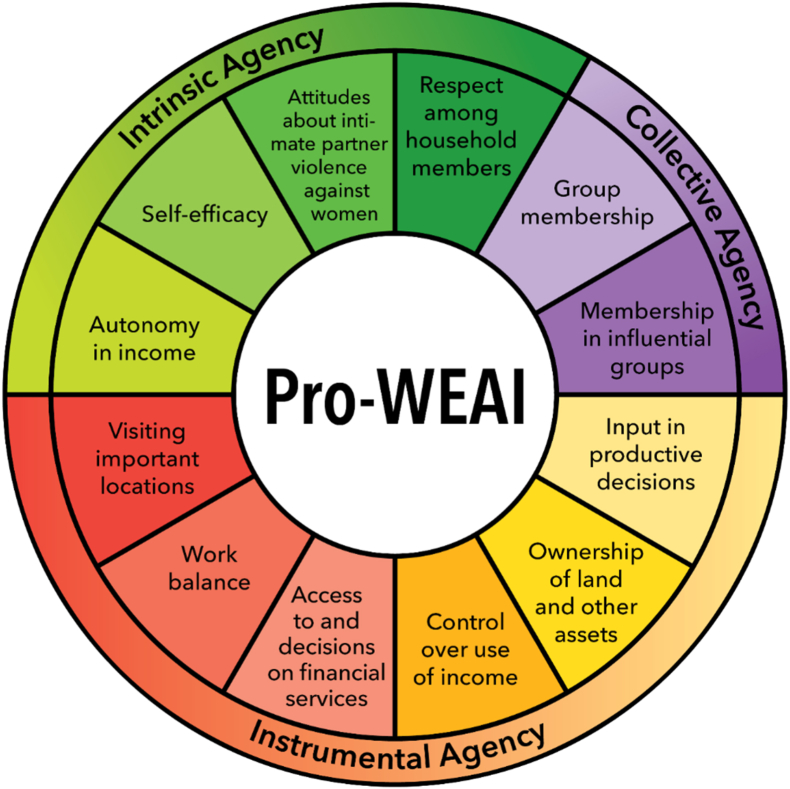
Domains and indicators of pro-WEAI.

Source: Authors

Pro-WEAI is the weighted sum of two subindexes: the Three Domains of Empowerment (3DE) and the Gender Parity Index (GPI), both calculated at the level of the sample or sub-sample. The 3DE assesses the degree to which women are empowered in three domains capturing intrinsic agency, collective agency, and instrumental agency. Pro-WEAI's intrinsic agency domain, or “power within,” comprises four indicators: (1) autonomy in income decisions; (2) self-efficacy; (3) attitudes towards intimate partner violence (IPV) against women; and (4) respect among household members. The collective agency domain, or “power with,” comprises two indicators: (1) group membership and (2) membership in influential groups. Finally, instrumental agency, or “power to,” has six indicators: (1) input in productive decisions; (2) ownership of land and other assets; (3) control over the use of income; (4) access to and decisions on financial services; (5) work balance; and (6) visiting important locations. The domains and indicators comprising pro-WEAI are presented in Fig. 1. Reflecting the process of co-development, pro-WEAI includes some indicators that are not in WEAI (self-efficacy, attitudes towards IPV against women, respect among household members, and membership in influential groups) and modifies the autonomy indicator in WEAI to focus on autonomy in the use of income, which is administered using vignettes instead of hypothetical questions. These modifications are mostly in the intrinsic agency domain (except for membership in influential groups in the collective agency domain).

The 3DE is constructed from individual-level empowerment scores, which reflect each person's achievements in the 12 equally weighted indicators. Each indicator measures whether an individual has surpassed a given threshold or has adequate achievement with respect to each indicator. In pro-WEAI, a woman is defined as empowered if she has adequate achievements in nine of the 12 indicators; the cutoffs defining adequacy are presented in Table 5 and are discussed more fully in Malapit et al. (2019). GPI compares the achievements of women and men within the same households.

**Table 5:** Definition of pro-WEAI indicators by type of agency measured.

Pro-WEAI Measure	Binary indicator	Continuous indicator
Pro-WEAI component indicators		
Intrinsic agency		
	Defined as adequate if:	
Autonomy in income	More motivated by own values than by coercion or fear of others' disapproval: Relative Autonomy Index score ≥ 1. RAI score is calculated by summing responses to the three vignettes about a person's motivation for how they use income generated from agricultural and non-agricultural activities (yes = 1; no = 0), using the following weighting scheme: 0 for vignette 1 (no alternative), −2 for vignette 2 (external motivation), −1 for vignette 3 (introjected motivation), and +3 for vignette 4 (autonomous motivation)	RAI score (ranging from 3 to −3)
Self-efficacy	‘‘Agree” or greater on average with self-efficacy questions: New General Self-Efficacy Scale score ≥ 32	Self-efficacy scale score (ranges from 8 to 40)
Attitudes about IPV against women	Believes husband is NOT justified in hitting or beating his wife in all 5 scenarios: 1) She goes out without telling him; 2) She neglects the children; 3) She argues with him; 4) She refuses to have sex with him; 5) She burns the food	Number of situations in which violence is not justified
Respect among household members	Meets ALL the following conditions related to their spouse, the other respondent, or another household member: 1) Respondent respects relation (MOST of the time) AND 2) Relation respects respondent (MOST of the time) AND 3) Respondent trusts relation (MOST of the time) AND 4) Respondent is comfortable disagreeing with relation (MOST of the time)	Number of conditions met from the following: 1) Respondent respects relation (MOST of the time); 2) Relation respects respondent (MOST of the time); 3) Respondent trusts relation (MOST of the time); 4) Respondent is comfortable disagreeing with relation (MOST of the time)
***Instrumental agency***		
	Defined as adequate if:	
Input in productive decisions	Meets at least ONE of the following conditions for ALL the agricultural activities they participate in: 1) makes related decision solely; 2) makes the decision jointly and has at least some input into the decisions; 3) feels could make decision if wanted to (to at least a MEDIUM extent)	Number of types of agricultural and non-agricultural activities for which the respondent makes decision solely, makes decision jointly and has at least some in input in the decisions, or feels could make decision
Ownership of land and other assets	Owns, either solely or jointly, at least ONE of the following: 1) At least THREE small assets (poultry, nonmechanized equipment, or small consumer durables); 2) At least TWO large assets; 3) Land	Number of asset types (including agricultural land) solely or jointly owned
Access to and decisions on financial services	Meets at least ONE of the following conditions: 1) Belongs to a household that used a source of credit in the past year AND participated in at least ONE sole or joint decision about it; 2) Belongs to a household that did not use credit in the past year but could have if wanted to from at least ONE source; 3) Has access, solely or jointly, to a financial account	Number of types of credit sources in which respondent participates in at least one sole or joint decision, plus access to sole or joint financial account
Control over use of income	Has input in decisions related to how to use BOTH income and output from ALL the agricultural activities they participate in AND has input in decisions related to income from ALL non-agricultural activities they participate in, unless no decision was made	Number of types of activities in which respondent has some control over use of income
Work balance	Works less than 10.5 h per day: Workload = time spent in primary activity + (1/2) time spent in childcare as a secondary activity	Time spent on paid and unpaid work, plus 0.5 x time spent on childcare as a secondary activity
Visiting important locations	Meets at least ONE of the following conditions: Visits at least TWO locations at least ONCE PER WEEK of [city, market, family/relative], or 2) Visits least ONE location at least ONCE PER MONTH of [health facility, public meeting]	Number of types of important locations visited
***Collective agency***		
Group membership	Active member of at least ONE group	Number of types of groups of which the respondent is an active member
Membership in influential groups	Active member of at least ONE group that can influence the community to at least a MEDIUM extent	Number of types of groups of which the respondent is an active member and which the respondent regards as influential
***Aggregate measures***		
Five Domains of Empowerment Index (5DE) (A-WEAI) or Three Domains of Empowerment Index (3DE)	Whether empowered: if individual achieves at least an empowerment score of 80% (A-WEAI) or 75% (pro-WEAI)	Empowerment score
Gender Parity Index (GPI)	Whether household achieves gender parity: woman's empowerment score is greater than or equal to the empowerment score of the male decision maker in her household.	Intrahousehold inequality score (men's empowerment score minus women's empowerment score)

Notes: There is a slight discrepancy in the definitions for the binary and continuous indicator for “input in productive decisions.” Projects calculated the original version of the binary indicator, which only included agricultural activities, whereas the continuous indicator was based on a revised version of the indicator, which includes both agricultural and non-agricultural activities. See Seymour et al. (2023) for more detail.

Our aggregate outcomes of interest are therefore defined as follows:

• Whether the individual is empowered, defined as achieving at least an empowerment score of 75% (binary).^9^
• Empowerment score, the weighted proportion of indicators in which a respondent is adequate (continuous).
• Whether the household achieves gender parity, meaning the woman is empowered or her empowerment score is greater than or equal to the empowerment score of the male decision maker in her household (binary).

Because pro-WEAI is a composite indicator, contrasting indicator-level impacts may cancel each other out in calculating the aggregate impacts. Owing to our focus on agency, we first estimate project impacts on adequacy in the 12 pro-WEAI indicators (using binary indicators) and on the variables that underlie the indicator itself (henceforth called continuous indicators).^10^ In this paper, we focus on impacts on the continuous indicators used to determine adequacy because binary indicators may be sensitive to the choice of thresholds or cutoffs. To account for differences in the scale and range of the continuous indicators, we estimate standardized coefficients or effect sizes, which involves scaling each coefficient by the standard error of the dependent variable.^11^ We then present impacts on the aggregate indicators, which comprise the three domains of empowerment captured by the 12 indicators. Since many of the projects had multiple treatment arms, each observation is the coefficient estimate of the specific treatment relative to the control. The domains and indicators of pro-WEAI are presented in Table 5.

## 4 Agency and empowerment impacts of the GAAP2 projects

We present quantitative and qualitative findings across the three types of agency—intrinsic, collective, and instrumental—that comprise pro-WEAI. For each type of agency, we first present the quantitative impacts on the continuous indicators and then interpret them in the light of the qualitative findings. In conducting the quantitative analysis, we first estimated impacts on binary indicators of adequacy^12^ and the continuous indicators underlying the binary indicators. Estimated impacts are found in Appendix Tables 6–9 for continuous indicators and in an online supplement for binary indicators, for both women and men. Comparison of the binary and continuous indicator estimates shows that the coefficients are quite similar in sign and statistical significance. For brevity, we focus on the continuous indicators, which may be more sensitive to incremental changes associated with program activities, and we graph the estimated coefficients. All impact estimates (effect sizes) are based on pro-WEAI, except for Heifer, which implemented A-WEAI, not pro-WEAI, and thus did not collect intrinsic agency indicators. Since all study participants in Heifer were members of groups in these group-based interventions, impacts on group membership, the only collective agency indicator in A-WEAI, were not estimated.

**Fig. 2:**
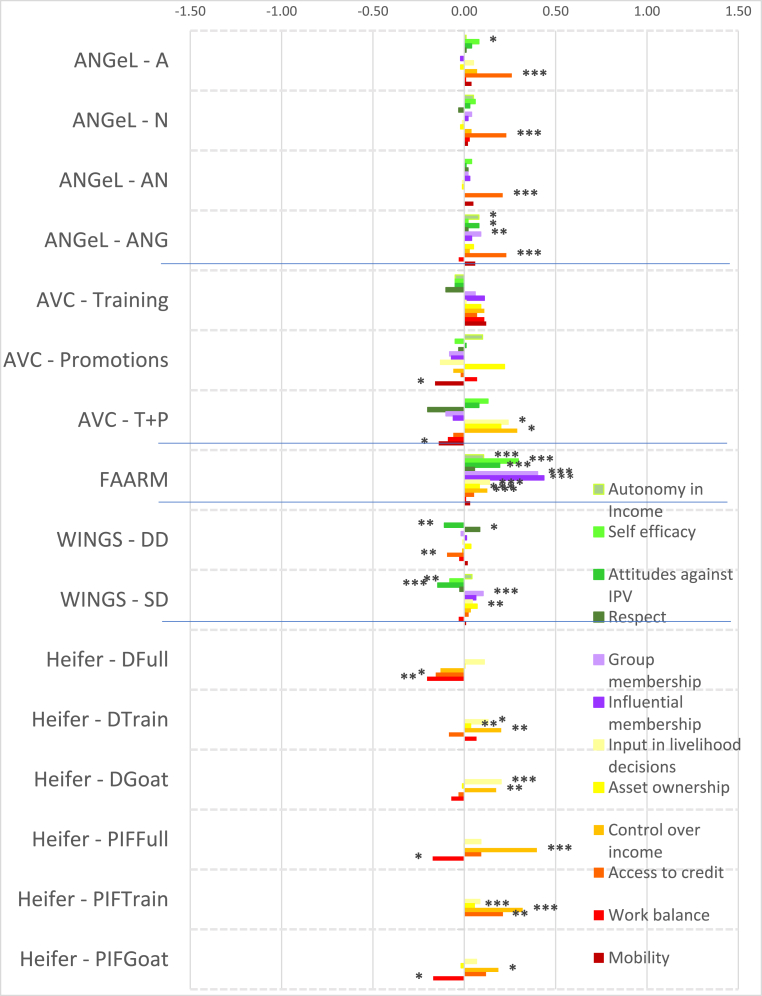
Estimated impacts on women's continuous indicators, South Asia projects, effect sizes Notes: *** significant at p < 0.01, ** significant at p < 0.05, * significant at p < 0.10. Note: See Table 4 for details on treatments.

**Fig. 3:**
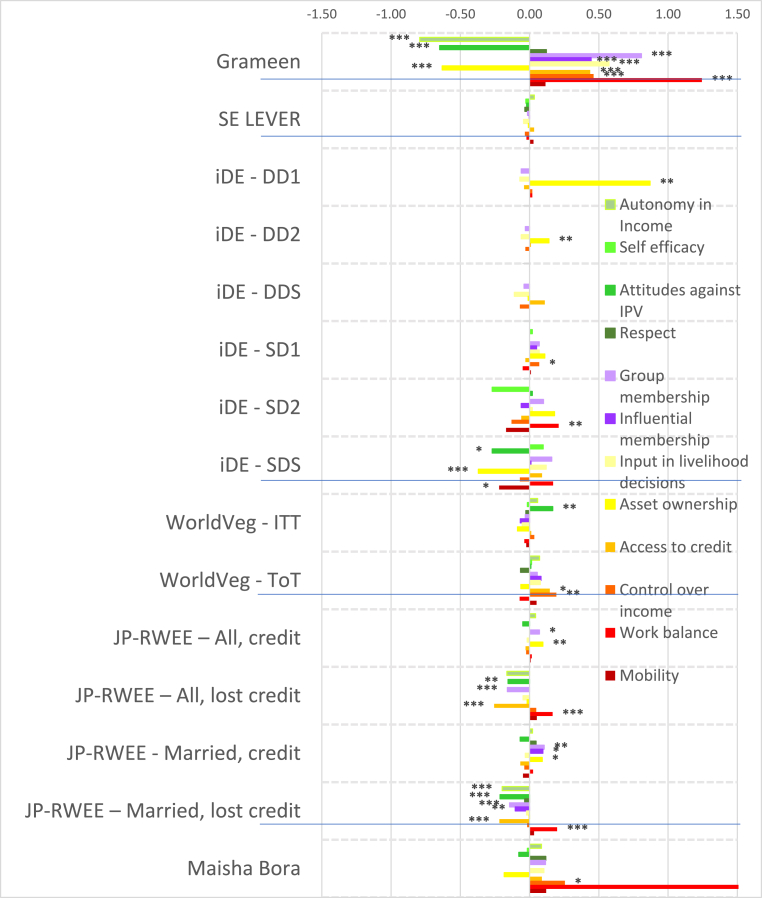
Estimated impacts on women's continuous indicators, Africa projects, effect sizes Notes: *** significant at p < 0.01, ** significant at p < 0.05, * significant at p < 0.10. Note: See Table 4 for details on treatments.

To account for the wide variation in agroecological conditions and gender norms across South Asia and Africa, we distinguish between the regions in presenting our results. Fig. 2 shows the magnitudes of the standardized impact coefficients on continuous indicators of women's agency, by treatment arm and estimation method, for the South Asia projects, and Fig. 3 for the Africa projects, based on estimated effect sizes in Appendix Tables 6 and 7 for women and men, respectively. Graphs of these effect sizes from the continuous indicator estimates are in standard deviation units, and the colors of each bar correspond to the type of agency in pro-WEAI: green for intrinsic agency, purple for collective agency, and orange for instrumental agency (see Fig. 1). Bars to the right of the vertical 0.0 line indicate positive impacts, those to the left signify negative impacts. Asterisks indicate whether the estimated impacts are statistically significant at the 0.01, 0.05, and 0.10 levels. Details on the treatment arm labels are presented more fully in Table 4.

Where possible, we use similar terminology for the indicators in discussing the quantitative and qualitative results. However, we recognize that the emic perspectives offered through qualitative methods—a strength of the approach—do not always map exactly to etic terms We do not standardize the language where quantitative and qualitative findings are different in cases where doing so would misrepresent respondents’ perspectives gleaned from qualitative approaches.

### 4.1 Impacts on intrinsic agency

Using the RBET framework, we expect that projects targeting transformation would address gender norms in their programming. Ten out of eleven projects (18 out of 32 treatments) addressed gender norms; only iDE did not include this in its programming. Some randomized controlled trials did not include changing gender norms in specific treatment arms, these were the treatments other than T-ANG in ANGeL, the NAAFCO promotions-only arm in AVC and some treatments in Heifer involving goat transfers without the pay-it-forward training. Given the importance that the projects themselves placed on normative change and their stated empowerment objectives, one would expect that intrinsic indicators would be affected.

#### 4.1.1 Quantitative findings

The quantitative findings identify the impacts on both women and men, although fewer projects collected the indicators of intrinsic agency for men.

##### 4.1.1.1 Impacts on women

Despite the majority of projects reporting transformative (T) objectives, the quantitative findings indicate that most treatments had overall null impacts on intrinsic agency indicators (green bars), although the few significant coefficients display some distinct regional patterns. The South Asia projects (Fig. 2) tend to have smaller dispersion (around zero) than the Africa projects (Fig. 3), and some of the Africa projects, notably Grameen (Crookston et al., 2021) and Maisha Bora (Krause et al., 2018), have noticeably large impact estimates, both positive and negative. Very few South Asia projects had significant impacts on autonomy with respect to income decisions, except for FAARM (Wendt et al., 2019; Waid et al., 2022), which found positive but small impacts.

We observe quantitatively larger but negative impacts on autonomy among the African projects. While none of the African projects demonstrate significant impacts on self-efficacy, positive impacts were also observed on FAARM beneficiaries in Bangladesh. Small negative impacts were observed in the nutrition-intensification WINGS intervention in India.

In contrast to the other intrinsic agency indicators, there were more significant impacts on attitudes towards IPV against women. In pro-WEAI, the attitude towards IPV indicator is constructed using respondents’ assertions that IPV against women is unacceptable in a series of circumstances.^13^ We interpret a larger number of circumstances deemed unacceptable as signifying greater empowerment. Neither FAARM nor WINGS had explicit activities targeting IPV towards women, but FAARM beneficiaries reported a small but positive increase in the number of incidents in which IPV is unacceptable, signifying higher levels of empowerment. In contrast, WINGS beneficiaries reported the opposite effects. WINGS did not have activities directly targeting men, but FAARM had household counseling visits (approximately every 2 months) and with the “lead farmer family” households.

There is a much wider range of impacts on attitudes towards IPV in the Africa projects than in the South Asia Projects (Fig. 3). WorldVeg beneficiaries reported that IPV against women is unacceptable in a greater number of instances, based on the list of five circumstances (Benali et al., 2020), whereas Grameen beneficiaries and JP RWEE beneficiaries who lost access to credit^14^ seemed to be willing to tolerate IPV against women under a wider range of circumstances (they report fewer unacceptable instances).

Finally, none of the projects in either South Asia or Africa exhibited significant impacts on respect within the household. The prevalence of null impacts on intrinsic agency indicators suggests that normative change may be slow. However, the increases in women's acceptance of IPV associated with empowerment-focused agricultural development programming is a matter of concern, which merits further qualitative investigation.

##### 4.1.1.2 Impacts on men

**Fig. 4:**
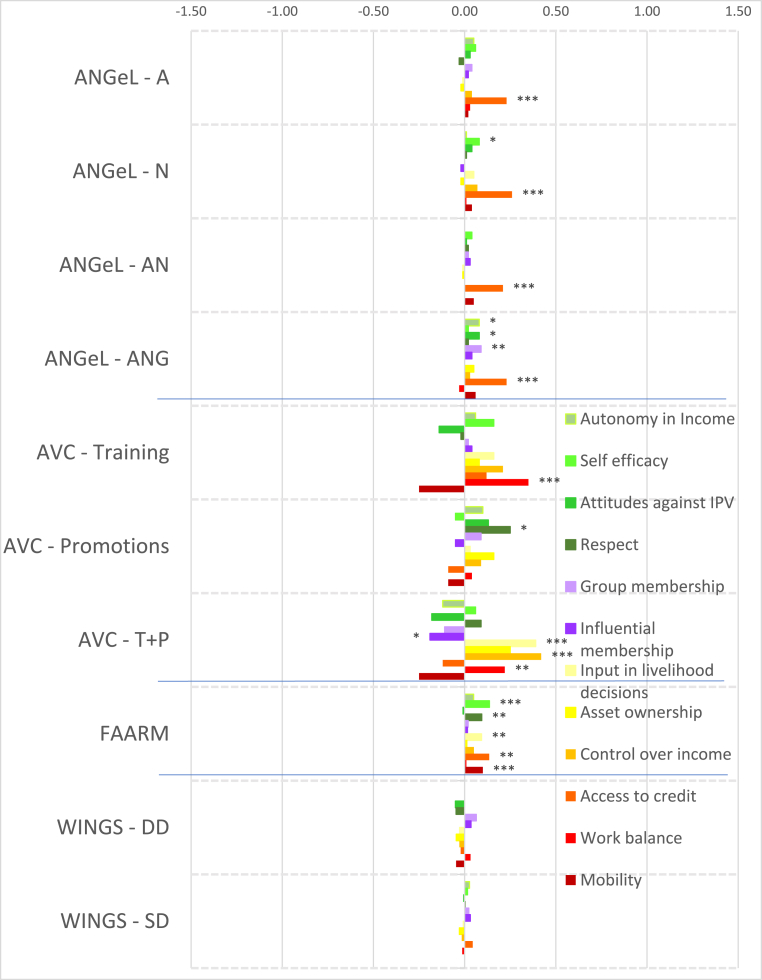
Estimated impacts on men's continuous indicators, South Asia projects, effect sizes. Notes: *** significant at p < 0.01, ** significant at p < 0.05, * significant at p < 0.10. Note: See Table 4 for details on treatments.

**Fig. 5:**
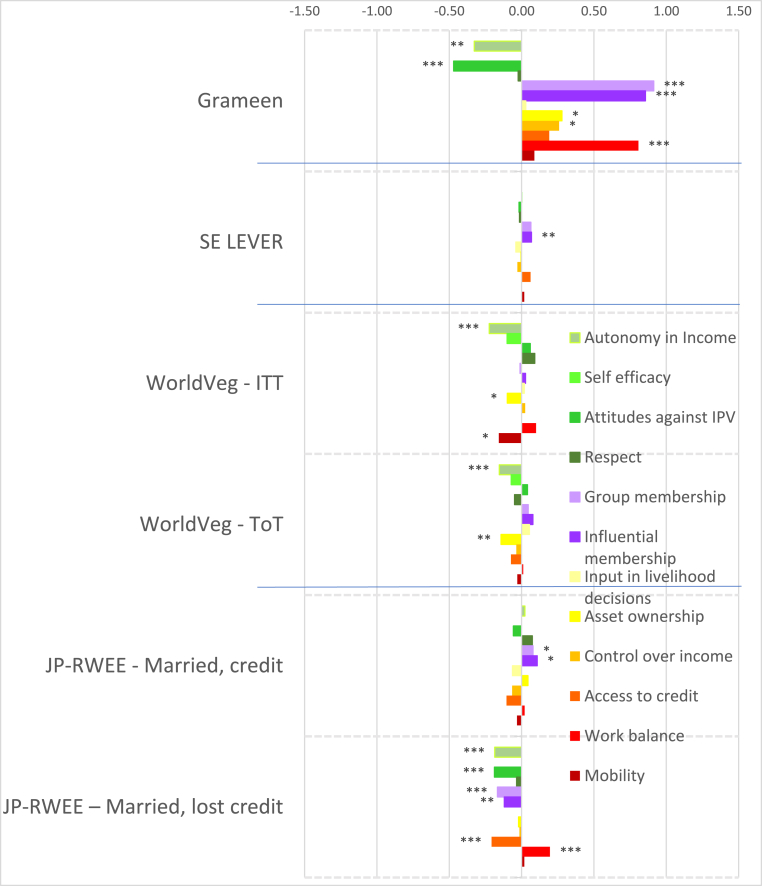
Estimated impacts on men's continuous indicators, Africa projects, effect sizes Notes: *** significant at p < 0.01, ** significant at p < 0.05, * significant at p < 0.10. Note: See Table 4 for details on treatments.

Fewer projects collected data on men's intrinsic agency indicators. Nevertheless, impacts on men tend to be smaller in magnitude, with fewer significant impacts (Fig. 4, Fig. 5). Only FAARM in Bangladesh had a small positive impact on autonomy with respect to income decisions (Fig. 4) but we detect negative and significant impacts on men's autonomy for Grameen beneficiaries and those who lost access to credit in JP RWEE, with relatively large estimated impacts for the latter, about 0.7 of a standard deviation (Fig. 5). Household dialogues such as in JP RWEE, which are approaches that aim to help husbands and wives identify common goals and approaches for achieving them, may introduce ideas of sharing decisions on income, which may have reduced men's feelings of autonomy in decision making.

Only FAARM improved men's self-efficacy. Interestingly, unlike the case for women, very few projects affected men's attitudes towards IPV against women. In both Grameen and JP RWEE, for those who lost access to credit, the intervention reduced the number of instances of IPV that men found acceptable. Across both South Asia and Africa projects, only FAARM had a positive impact on respect within the households (albeit a small impact); other impacts on men were null. The prevalence of small and/or null impacts suggests the difficulty of changing norms and attitudes through agricultural development programming within the limited time frame of typical impact evaluations.

#### 4.1.2 Qualitative findings

Contrary to the prevalence of null impacts in the quantitative findings, qualitative findings indicate that the projects affected several aspects of intrinsic agency. The qualitative studies elicited a broad range of examples from participants about how their self-confidence—a component of self-efficacy--increased, which they attributed directly to the program activities. FAARM, which provided women with gardening materials and trainings, found that they often sold the surpluses of the vegetables they produced and maintained control over the income generated, either saving it or spending it on personal needs, family needs, additional food, or their children's education (Dupuis et al., 2022). FAARM beneficiaries reported greater confidence and motivation when they saw their gardens becoming productive (Dupuis et al., 2022). In JP RWEE, the opportunity for women to earn money helped them develop a positive self-image and be proactive about their rights and responsibilities. SELEVER beneficiary women said raising poultry increased their self-confidence in their skills and capacities. For these women beneficiaries, gaining financial independence was critical, as it helped them to rely less on their husbands' permission or direction for how to spend money (Eissler et al., 2020).

Capacity building strategies may have been important to strengthening women's intrinsic agency, even if they ostensibly directly targeted instrumental agency. Findings from seven qualitative studies (ANGeL, FAARM, Heifer, Grameen, SELEVER, WorldVeg, JP RWEE), reveal that beneficiaries perceive capacity building projects as having a strong, positive influence on their self-efficacy. Overall, many women beneficiaries described feeling more confident, and directly attributed their increased confidence to the program activities. Notably, women beneficiaries in the Grameen project shared that their participation in a savings group encouraged norm change at the community level around women's ability to contribute to household income (Kieran et al., 2018). Additionally, ANGeL participants noted that due to their increased knowledge stemming from the training activities, others in the community would seek them out for advice (Quisumbing et al., 2021). This matches the emic notions of women's empowerment stemming from being able to do things for others (Meinzen-Dick et al., 2019).

The findings on attitudes towards IPV deserve a closer look, particularly in those projects that reported a positive (quantitative) impact. Qualitative work on FAARM conducted at endline suggests that the effect on attitudes towards IPV was an ancillary benefit of improved gender equality resulting from the combined activities. Dupuis et al. (2022) included findings from men and women who said that women's status in the household had improved because of the intervention; as women's status improves, one would expect to see fewer men and women agreeing that IPV is acceptable. Moreover, in in the Sylhet division where the study took place, women's mobility is quite low, and some households did not want the women to attend program meetings alone. So FAARM encouraged family units—the woman with her spouse or another family member—to attend. It is possible that, because of this, FAARM had a more holistic reach than programs in areas where it was not a problem for women to attend alone. In contrast, programming in WINGS was targeted only to the woman in the household. Kumar et al. (2021, citing (de Hoop et al., 2014); (Jakimow and Kilby, 2006) acknowledge the limitations of women's SHG approaches since they engage only one side of the gender equation in contrast to those approaches that reach both women and men.

Although we did not detect any significant impacts on the “respect within the household” quantitative indicator (possibly because “respect” may be expressed differently in different contexts) the qualitative work reveals some of the subtleties in unpacking impact on intrahousehold dynamics. We find that intrahousehold dynamics are complex, varying from context to context, and may be a constraint to changing gender norms. For instance, from the Grameen project in Burkina Faso, we found that both women and men believe women should be “submissive” to their husbands to show appropriate deference, though women and men both perceive “emancipated” women positively. If such projects target their messages mainly to women, they could weaken the potential to change men's attitudes. Our qualitative findings take this concept further by prompting us to consider household structures beyond the nuclear structure and take an intersectional approach. For instance, in polygynous contexts like the Maisha Bora project, women may perceive that their husband treats them differently than he does a co-wife, which may lead to tension among women in the household. In the Heifer project in Nepal, we found that women who live with their mothers-in-law have little control over their time and responsibilities, as their lower social status in the household restricts them from exercising greater autonomy.

### 4.2 Impacts on collective agency

Nine out of 11 projects (25 out of 32 treatments) attempted to strengthen organizations, which, in this context, means strengthening women's groups. In our strategy-to-agency mapping (Table 4), this translates to efforts to increase collective agency, which lies squarely in the “empower” objective in the RBET framework. The only two projects without an explicit objective to strengthen group functioning were ANGeL and AVC, both in Bangladesh, although AVC initially delivered some programming through NGOs.

#### 4.2.1 Quantitative findings

##### 4.2.1.1 Impacts on women

Given that many programs worked through women's groups, one might expect that projects would have positive impacts on collective agency indicators (purple bars in Fig. 2, Fig. 3). However, estimated impacts are mostly null. It is possible that selected interventions were implemented among women who were already members of groups, so the intervention would not necessarily have affected the number of types of groups or influential groups to which women belonged. That is, if the comparison group consisted of women who already belonged to groups, no differential impact may have been detected. We notice positive impacts on both the types of groups and influential groups to which women belonged in Grameen, which aimed to establish women's savings groups, and on JP RWEE members who retained access to credit through groups formed as part of the program. Those JP RWEE beneficiaries who lost access to credit unsurprisingly experienced negative impacts on both the number of types of groups to which they belonged and the number of types of influential groups.

##### 4.2.1.2 Impacts on men

We did not expect to see many significant impacts on men's collective agency (purple bars in Fig. 4, Fig. 5), given the focus of programming on women's-groups in the portfolio. Nevertheless, a few projects had impacts on men's collective agency indicators. The gender sensitization arm of ANGeL (T-ANG) had the lone significant (though small) positive impact on the number of types of groups to which men belonged among the Asian projects, even if strengthening organizations was not an explicit objective of the project, and interestingly, so did Grameen, among the African projects. Men in Grameen beneficiary households also experienced positive impacts on the number of types of influential groups to which they belonged. However, men in households of JP RWEE beneficiaries who lost access to credit because their wives left the savings group or could not repay their loans experienced negative impacts on the number of types of groups to which they belonged.

#### 4.2.2 Qualitative findings

Unlike the quantitative findings, the qualitative studies found positive associations between projects and perceptions of increased collective agency. The qualitative study with Maisha Bora found that women perceive group membership as key to empowerment, though lack of spousal support often prevents women from participating (Krause et al., 2018). AVC participants perceive group membership as offering many benefits, though inconvenient timings and locations make group participation challenging for some (Rubin et al., 2018). Qualitative work associated with Heifer shows that, because groups reproduce power relations and exclusion from broader Nepali society, women with less education or status may lack the confidence to speak in groups or fully benefit from them (Nepā School of Social Sciences and Humanities, 2017). Qualitative research on WINGS found that groups with stronger existing relationships with the implementing organization were better able to engage and benefit their members. Past associations with PRADAN that led to benefits helped to build trust and strengthen participation not seen in newly formed groups (Nichols, 2021).

The qualitative studies that examined perceptions of group membership (FAARM, Heifer, JP RWEE, and Maisha Bora) affirm the improvements in collective agency, and interlinkages with other aspects of empowerment. In select cases, women's experiences spoke directly to the benefits of collective agency. For instance, in Nepal (Heifer), one woman shared a story about how her fellow group members came to her home to humiliate her husband for beating her, an event which halted any potential future violence (Nepā School of Social Sciences and Humanities, 2017). In Bangladesh (FAARM), beneficiaries who experienced the greatest gains in agency attributed it, at least in part, to support from other women who were fellow group members (Dupuis et al., 2022). In some cases, women's group members shared surplus agricultural inputs amongst themselves in lieu of selling them, affirming their social relationships and demonstrating how the benefits of collective agency could facilitate women's input into productive decisions or control over agricultural produce. Indeed, because so many projects use groups to deliver programs, there are two-way relationships between collective agency and program effectiveness. The JP RWEE, Maisha Bora, and WINGS qualitative studies showed that constraints to participation in groups, such as a lack of spousal support, transportation, or time poverty (which are aspects of instrumental and intrinsic agency) limited the participation of some women in the overall project.

### 4.3 Impacts on instrumental agency

All 11 projects had either capacity building objectives (9 of 11 projects or 68.8% of treatment arms) or aimed to provide goods and services (8 out of 11 projects or 65.6% of treatments). With all projects targeting some aspect of instrumental agency to meet a combination of reach, benefit, and empower objectives, we expect to detect impacts on instrumental agency indicators.

#### 4.3.1 Quantitative impacts

The instrumental agency domain has the largest numbers of indicators in pro-WEAI, reflecting projects’ interest in these indicators, and, by construction, contributes most to the empowerment score. Estimated impacts on the continuous indicators are in Appendix Tables 8 and 9 for women and men, respectively. Estimated impacts are indicated by yellow, orange, and red bars in Fig. 2, Fig. 3, Fig. 4, Fig. 5.

##### 4.3.1.1 Impacts on women

In contrast to the intrinsic agency indicators, we observe a larger number of significant impacts on instrumental agency indicators across projects in terms of number and magnitude of effect sizes (Fig. 2). Among the South Asia projects, FAARM and the Heifer treatment arm that distributed goats had positive and significant impacts on the number of types of productive decisions that women made; among the Africa projects, Grameen similarly increased the number of types of productive decisions made. The WINGS project, FAARM, and the Heifer-values based treatment arm (DTrain) all significantly increased the number of types of assets that women owned. The iDE irrigation pumps project in Ghana unsurprisingly increased the number of types of assets owned, since this project aimed to increase the use of small-scale irrigation pumps, but negative effects were observed on spillover communities (treatments coded S) (Fig. 3). While beneficiaries who retained access to credit in JP RWEE increased the number of types of assets owned, Grameen beneficiaries experienced a negative impact on the number of types of assets owned. This finding could, however, reflect a consolidation of asset portfolios into fewer, more valuable assets, and is a drawback of this indicator.

Interestingly, many projects that did not explicitly include credit provision had positive impacts on the number of types of credit decisions made. These include all treatment arms of ANGeL and the “pay-it-forward” (PIF) beneficiaries in the Heifer values-based treatment arm. Although the WINGS intervention reported a small negative impact on the number of types of credit decisions made, this is not robust to type of estimation procedure.^15^ Among the Africa projects, the Grameen program, a savings and credit intervention, unsurprisingly had positive impacts on the number of types of credit decisions that women made. Women who lost access to credit in JP RWEE experienced negative impacts on the number of types of credit decisions that they made, another expected result.

Several treatments in the South Asia portfolio increased women's control of income, measured by the number of types of income decisions they made. These include FAARM, Heifer direct beneficiaries (values-based and goat distribution programs), and Heifer PIF beneficiaries (full treatment and values-based program). Among the Africa projects, we observe positive impacts on the number of types of income decisions made in the Grameen and WorldVeg projects.

Whether agricultural development projects increase women's workload is an important concern. This concern appears to be unfounded in the South Asia projects (some Heifer treatment arms even reduced work hours, while the others had null effects), but is justified in the Africa projects. Women in the Grameen project, those who lost credit in JP RWEE, and those with motor pumps in the small-scale irrigation project in Ghana experienced increases in workload. This compounds the already high workload of women in African agriculture. Finally, none of the projects significantly affected the number of types of important places that women visited, an indicator of mobility.

##### 4.3.1.2 Impacts on men

Although these projects had women's empowerment objectives, it is worthwhile to examine their impacts on men (Fig. 4, Fig. 5) for indications of men appropriating benefits, spillovers that also help men (e.g. if they listened in or took fair advantage of a new service in the community), or negative spillovers, particularly if no positive impacts were detected on women. Only the AVC training plus promotions treatment and FAARM had positive impacts on the number of types of productive decisions that men made (no similar effects were observed on women in AVC), while no significant effects were detected for the Africa projects (Fig. 4). No significant impacts were detected on the number of types of assuets that men owned, except for a small negative impact in WorldVeg. All the ANGeL treatment arms and FAARM increased the number of types of credit decisions that men made (similar to the effects on women); among the Africa projects, only men in JP RWEE beneficiary households that lost access to credit experienced negative impacts.

Interestingly, men in the AVC (treatment plus promotions program) experienced increases in the number of types of income decisions made without any corresponding impacts on women in that program; no significant impacts were detected among the Africa projects. However, the positive impacts in AVC seem to have occurred at the expense of increasing men's workload. Workload also increased for men in the Grameen project, and for men in JP RWEE who lost access to credit. Finally, only FAARM increased the number of types of important places that men visit; all other projects had null impacts.

#### 4.3.2 Qualitative findings

Consistent with many South Asia projects documenting positive impacts on instrumental agency, the qualitative work confirmed that ANGeL and FAARM respondents perceived women's participation in agricultural decision making had increased over the course of the project (Dupuis et al., 2022). However, in AVC, associated qualitative work confirmed that gender norms still favor men's control over productive decisions. In WINGS, qualitative research revealed that women's participation in the self-help groups through which nutrition education was offered was not equally accessible to all participants. Attendance, and thus access to some benefits, was constrained by workloads and lack of spousal support (Nichols, 2021).

In Ghana, iDE beneficiaries, who received irrigation technology, acknowledged that women's control over the income they generate varies from family to family (Bryan and Mekonnen, 2022). Some ambivalence is evident in the Maisha Bora context, where women typically have little control over decision making of any kind. Having a business bolsters women's control over income but may threaten some notions of masculinity, as women need permission from their spouses on nearly everything (Krause et al., 2018).

In the qualitative studies of the Grameen, JP RWEE, and Heifer projects, all of which employed capacity building strategies, both women and men perceived women as having greater access to credit relative to men due to the increasing popularity of microfinance groups targeting women, even though some projects (Heifer) did not offer credit. These three projects were not the only interventions in their respective areas, and capacity building activities may have helped women tap into credit available from other sources. However, the qualitative work also finds that spousal approval, greater access to transportation, and shorter travel times enhance women's freedom of movement (to participate in community groups, including but not limited to credit groups) and women's access to credit (Meinzen-Dick et al., 2019).

Qualitative work also provides insight into decision making on credit and financial services. There is strong evidence that joint decision making is desirable among women and men, though the meaning of jointness varies across contexts (Meinzen-Dick et al., 2019). In some contexts, women's decision making is perceived as threatening to masculinity, though women report their spouses consulting them before taking a decision (Meinzen-Dick et al., 2019). As previously discussed, spousal approval may dictate women's access to credit, either in terms of women needing to seek permission to participate in credit groups or men exerting undue influence on how borrowed funds should be spent.

Regarding freedom of movement, many women reported needing their husbands' permission for both travel and/or participation in community groups, ranging from sharing one's purpose for traveling (SELEVER) to negotiating one's absence (Heifer, SELEVER, Grameen, WorldVeg). In an extreme case, Maasai women in the Maisha Bora project in Tanzania shared that their spouses may beat them to prevent them from traveling or participating in groups (Krause et al., 2018). Conversely, qualitative respondents for the Heifer project in Nepal attributed their greater freedom of movement to their involvement in credit groups, which families encouraged because they saw benefits for the whole family (Nepā School of Social Sciences and Humanities, 2017). In the Grameen and SELEVER studies, women acknowledged they may borrow from credit groups only to allow their husbands to control how the funds are used (Kieran et al., 2018; Eissler et al., 2020).

Despite the strong qualitative evidence on the links between freedom of movement and access to credit, in some contexts, the qualitative work also unexpectedly found that capacity building strategies may allow beneficiaries to avoid taking credit in specific circumstances. For instance, in SELEVER, which supported women's poultry raising, women reported they no longer needed to take credit to purchase meat to serve to visitors or during celebrations; they could slaughter one of their own chickens (Eissler et al., 2020). As such, reductions in women's credit sources may indicate that projects have helped women acquire the resources needed to leverage other, more preferable, choices around credit, which is not inherently disempowering.

#### 4.3.3 Summary of findings on continuous indicators

Fig. 6a, Fig. 6b summarize the distribution of impact estimates across all 12 pro-WEAI indicators for each of the treatment arms for women and men, respectively. These distributions are presented both in absolute terms (counts of the negative, null, or positive estimates) and in percentages. Confirming the plotted effect sizes, most estimated impacts for men and women are null. The largest number of estimated positive impacts are observed among instrumental agency indicators and collective agency indicators, the latter reflecting group-based programming, Intrinsic agency indicators are the least affected by the agricultural development projects in the GAAP2 portfolio. The prevalence of null impacts among the intrinsic agency indicators is not simply because fewer projects collected these indicators; this result holds when we examine percentage distributions.

**Fig. 6a:**
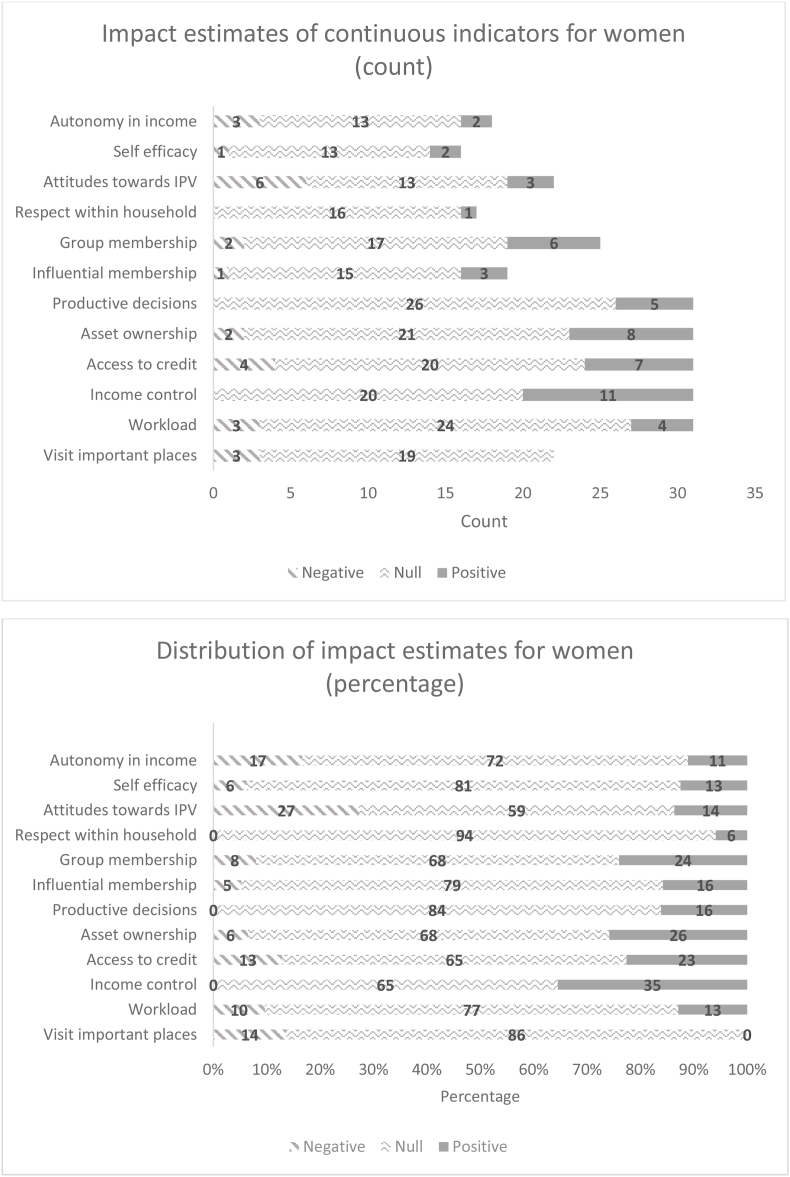
Distribution of impact estimates for continuous indicators, women.

**Fig. 6b:**
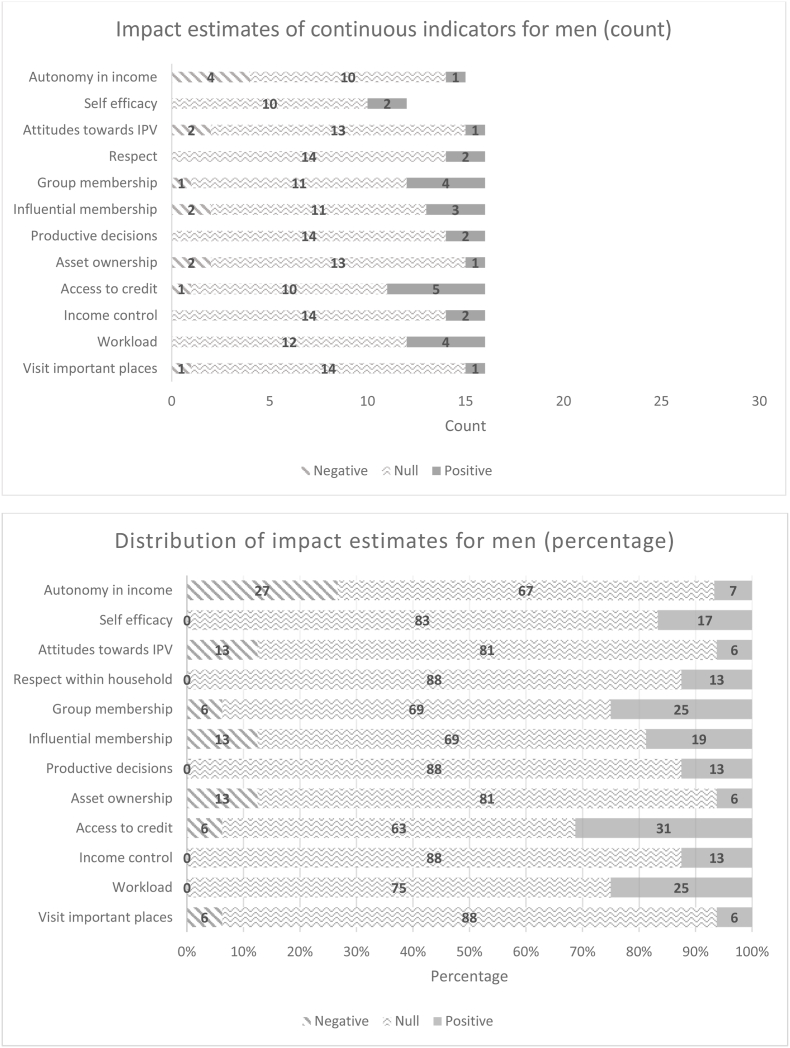
Distribution of impact estimates for continuous indicators, men

The different findings reached using qualitative and quantitative methods may reflect both the sensitivity of the quantitative tool and the possibility that pro-WEAI is not measuring the right constructs. This dissonance may be more relevant to the intrinsic agency indicators, where women may self-identify aspects of intrinsic agency that are not anticipated and captured in the standardized quantitative questionnaires. Moreover, aspects of intrinsic agency are linked to gender norms, which may be slower to change. Because quantitative questions are pre-specified and follow-up questions in qualitative work can adjust to the answers of respondents themselves in real time, the latter may serendipitously unearth aspects of intrinsic agency that were not part of the quantitative instrument.^16^ These answers provide valuable information on how projects are being perceived, even if these perceptions are not expressed in common terms across many participants. Moreover, even when the quantitative instrument is adapted based on exploratory work at baseline, there may be impacts that are identified in the qualitative work that could not have been anticipated and included in the survey instrument. Even excellent exploratory work may not predict what the impacts may be in advance.

Similarly, the richness of the findings from the qualitative work on collective agency and the limitation of having only two quantitative collective agency indicators in pro-WEAI suggest that better or additional measures of collective agency are needed. The existing quantitative indicators would not be able to measure group quality. Moreover, it may take time for a group to form, and even longer for it to be seen as influential, which means that the impact would extend beyond the timeline of the project.

Pro-WEAI detected more impacts on instrumental agency, possibly because projects may be more likely to have tested strategies that directly target instrumental agency through their capacity building and goods/services provision strategies (Table 4 indicates that 9 and 8 out of 11 projects, respectively, have these types of strategies). Even if more projects claim to have strategies targeting gender norms, those strategies may be newer or may be ineffective in their particular context.

### 4.4 Overall impacts on aggregate indicators

Fig. 7a, Fig. 7b are bar charts showing estimated project impacts on the aggregate women's empowerment measures (whether empowered and the empowerment score) in South Asia and Africa projects, respectively, and Fig. 8a, Fig. 8b are the corresponding bar charts for men. Fig. 9 shows the distribution of these coefficients according to whether these impacts were positive, negative, or null (at p < 0.05). Fig. 10a, Fig. 10b shows bar charts with the estimated project impacts on the probability that a household attains gender parity for South Asia and Africa projects, respectively. Finally, Fig. 11 presents the distribution according to whether impacts on gender parity were positive, negative, or null.

**Fig. 7a:**
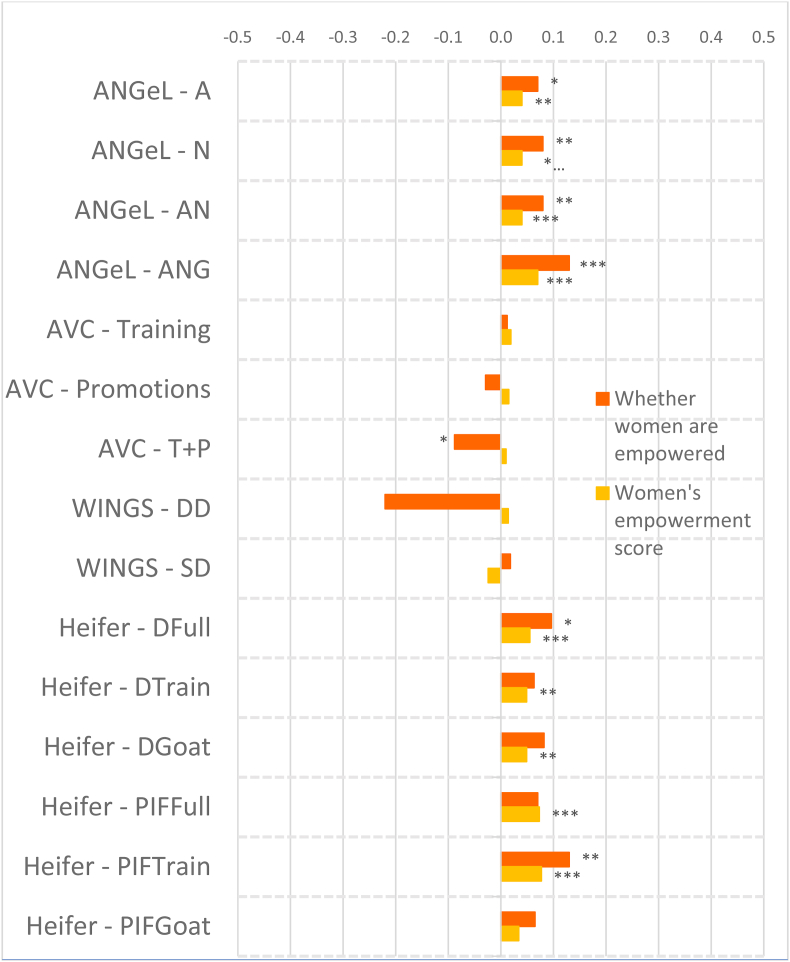
Estimated impacts on composite indicators of women's empowerment, South Asia projects Notes: *** significant at p < 0.01, ** significant at p < 0.05, * significant at p < 0.10. Excludes FAARM, which estimated odds ratios. See Table 4 for details on treatments.

**Fig. 7b:**
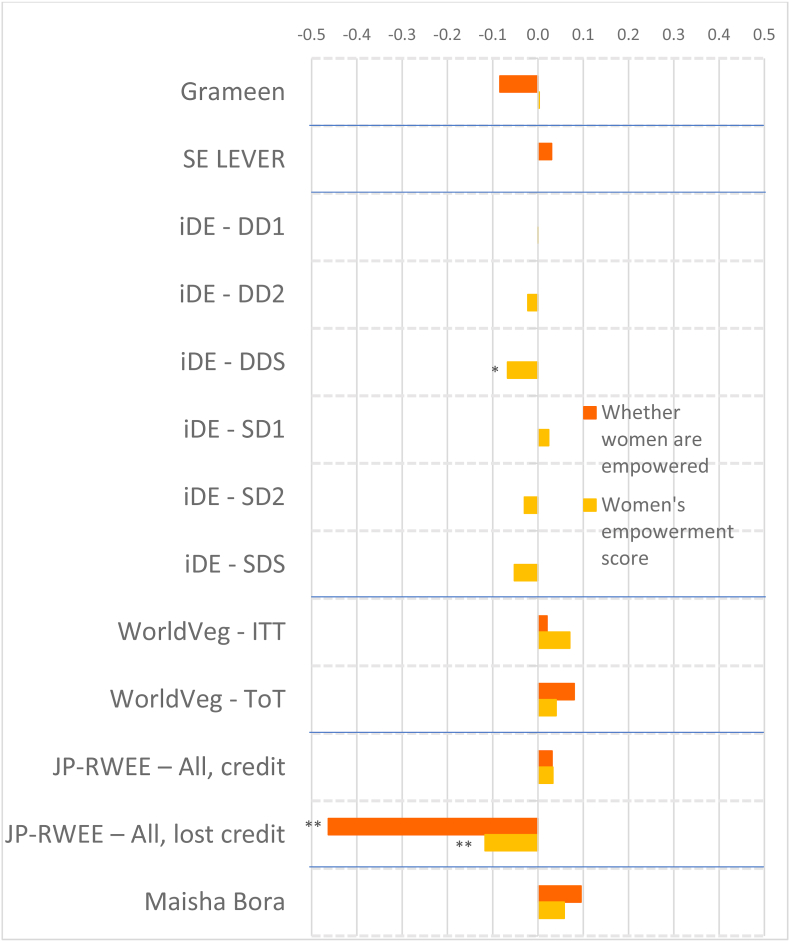
Estimated impacts on composite indicators of women's empowerment, Africa projects Notes: *** significant at p < 0.01, ** significant at p < 0.05, * significant at p < 0.10. See Table 4 for details on treatments.

**Fig. 8a:**
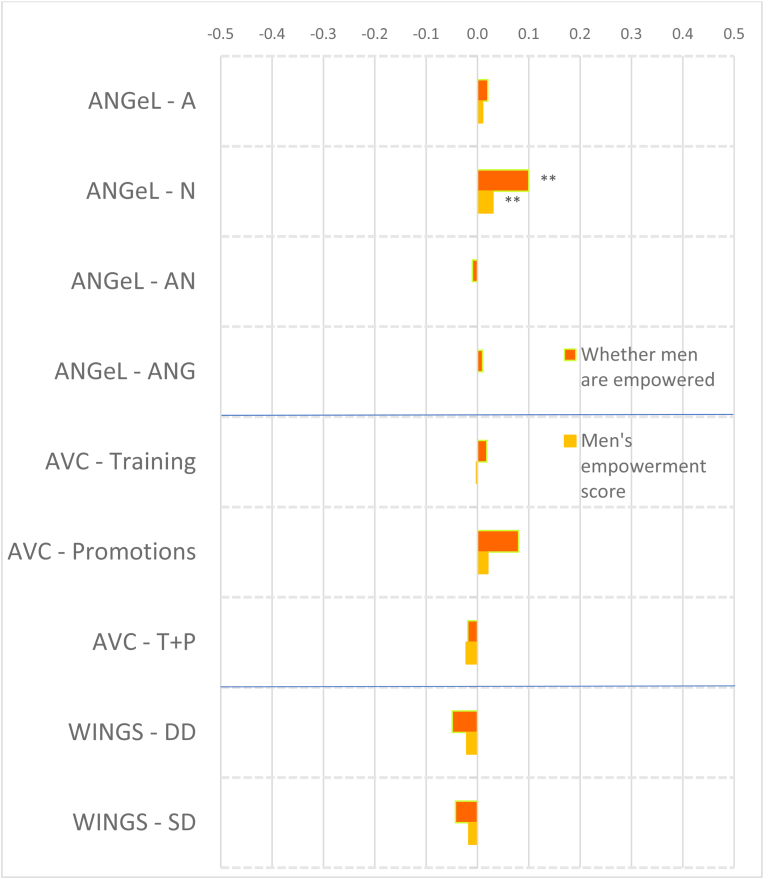
Estimated impacts on composite indicators of men's empowerment, South Asia projects Notes: *** significant at p < 0.01, ** significant at p < 0.05, * significant at p < 0.10. Excludes FAARM, which estimated odds ratios. See Table 4 for details on treatments.

**Fig. 8b:**
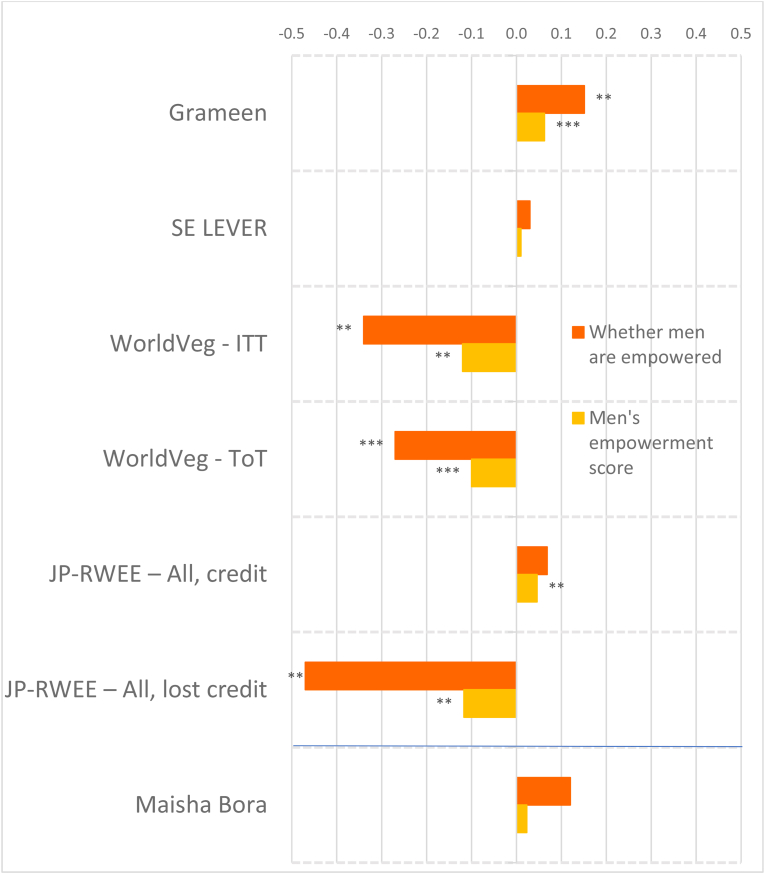
Estimated impacts on composite indicators of men's empowerment, Africa projects Notes: *** significant at p < 0.01, ** significant at p < 0.05, * significant at p < 0.10. See Table 4 for details on treatments.

**Fig. 9:**
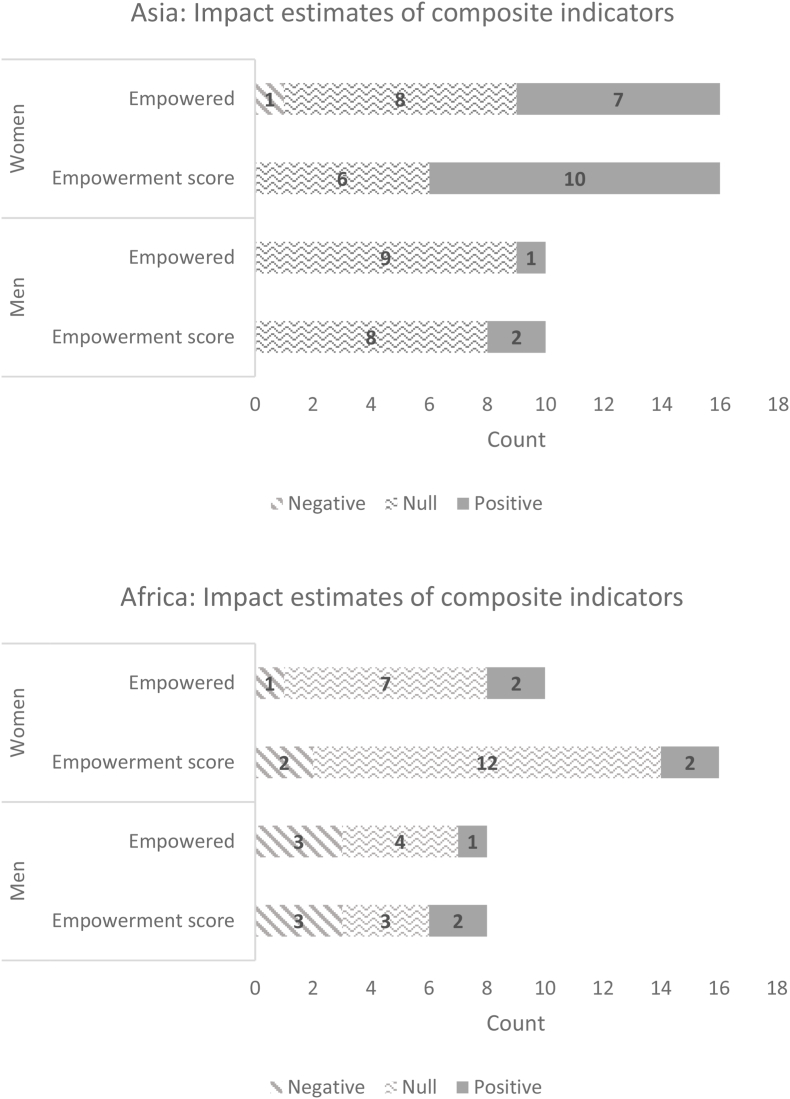
Distribution of impact estimates on whether empowered and empowerment score Number of estimated coefficients: Asia: Women: 16; Men: 10; Africa: Women: 16; Men: 8. Count refers to the number of estimated impact coefficients across treatment arms in the GAAP2 portfolio (where measured); includes FAARM. Definition of variables: Empowered denotes whether the individual is empowered (binary): An individual is defined as empowered if they achieved at least an empowerment score of 80% (A-WEAI) or 75% (pro-WEAI) Empowerment score (continuous): This is the proportion of indicators in which a respondent is adequate.

**Fig. 10a:**
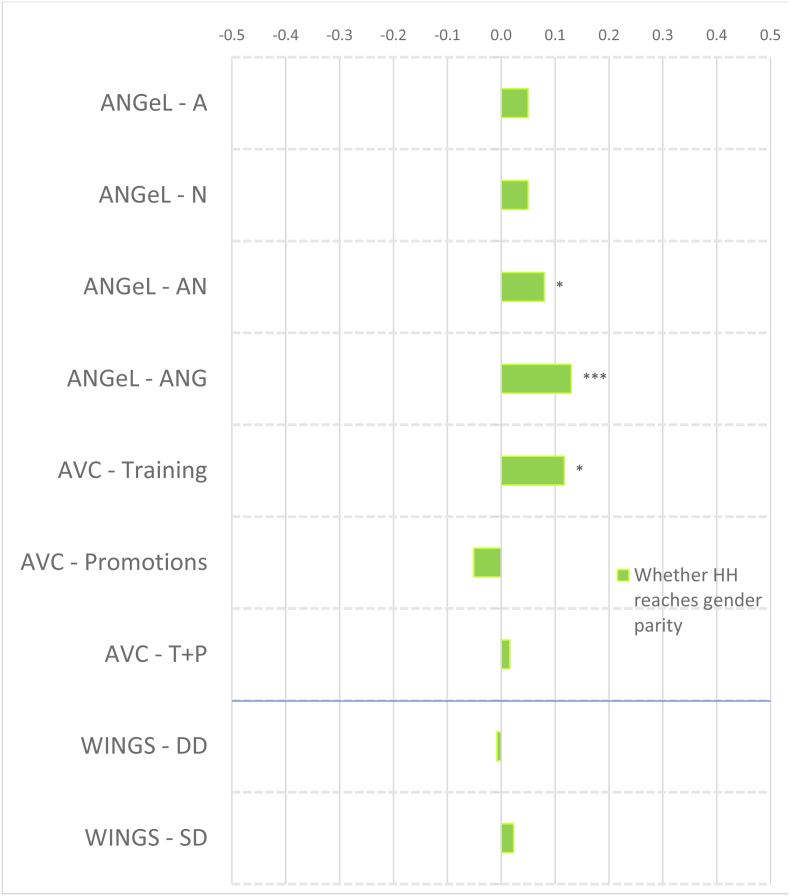
Estimated impacts on probability that household attains gender parity, South Asia projects Notes: *** significant at p < 0.01, ** significant at p < 0.05, * significant at p < 0.10. Excludes FAARM, which estimated odds ratios, and Heifer, which did not collect data on men. See Table 5 for details on treatments.

**Fig. 10b:**
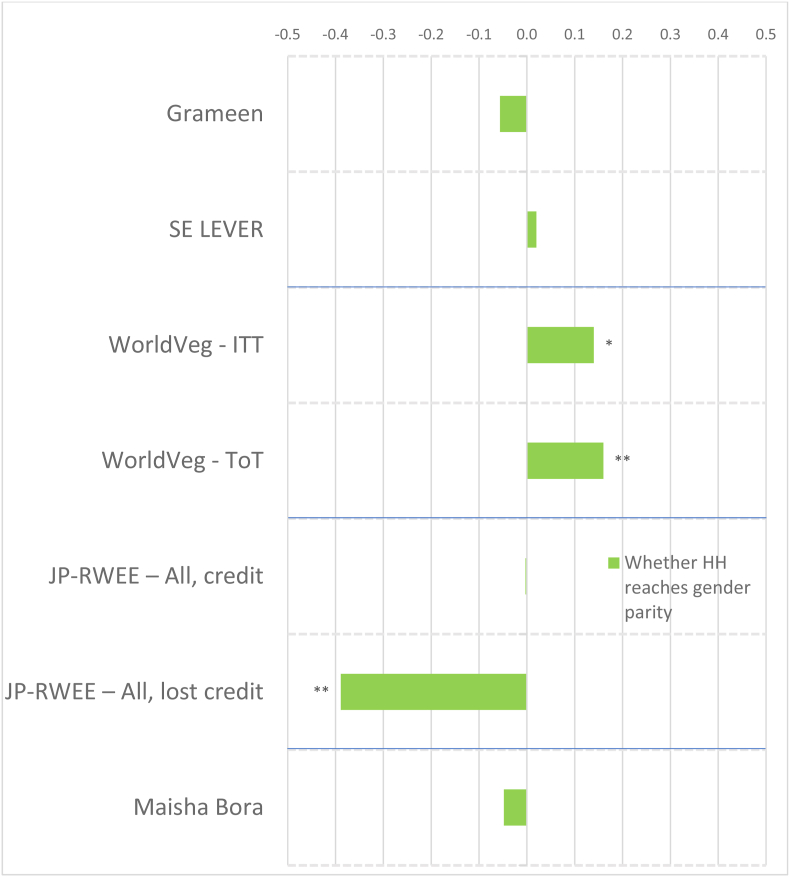
Estimated impacts on probability that household attains gender parity, Africa projects Notes: *** significant at p < 0.01, ** significant at p < 0.05, * significant at p < 0.10. See Table 5 for details on treatments.

**Fig. 11:**
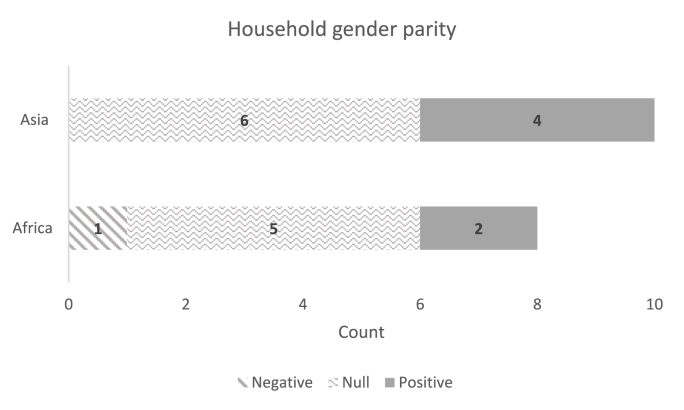
Distribution of impact estimates on whether the household achieved gender parity Notes: Count refers to the number of estimated impact coefficients across treatment arms in the GAAP2 portfolio (where measured). Definition of variables: whether the household achieved gender parity.

Given the prevalence of null impacts on the component indicators, it is unsurprising that most projects overall did not have a significant impact on the aggregate empowerment indicators. Of the 32 treatment arms across the 11 projects, there are 9 and 12 cases of significant positive impacts on whether the woman is empowered and the women's empowerment score, respectively, and 2 cases of negative impacts for both measures. In contrast, there are 15 and 18 cases of insignificant or null results on these indicators, respectively.

Disaggregating by region, the positive cases are heavily concentrated in South Asia, where 7 of 16 treatment arms are associated with empowerment, and a majority (10 of 16) cases had significant increases in empowerment scores. The positive impacts on aggregate measures for women are found in all the ANGeL and Heifer treatment arms; negative impacts on overall empowerment are experienced by women in JP RWEE who lost access to credit. The FAARM project, which estimated odds-ratios for empowerment status, also had a positive impact on women's aggregate empowerment measures. There are fewer significant impacts reported for men, which is not surprising since some projects did not collect data on men and most projects did not target men's empowerment. Men in the ANGeL nutrition treatment arm and Grameen experienced improvements in empowerment, but those in WorldVeg, and the JP RWEE group that lost access to credit had worse empowerment outcomes. Similar to the component indicators, there is less variation in the impact estimates in South Asia compared to Africa. Most of the Asian projects were nutrition-sensitive agricultural projects that shared similar design features (e.g., encouraging homestead gardening or livestock raising). In contrast, the Africa projects were not only more diverse in terms of program components (credit, home gardens) but also with respect to the cultural norms and livelihoods of the targeted West, East, and Southern African populations.

Very few projects reported significant quantitative impacts on gender parity (Fig. 11). Out of 10 treatment arms in South Asia that measured gender parity, 4 reported improvements, and 6 reported null impacts. Among 10 treatment arms measuring gender parity in Africa, only 2 reported improvements, 5 reported null impacts, and 1 reported a deterioration. Improvements in gender parity occurred in FAARM, the T-ANG treatment arm of ANGeL, and WorldVeg (in the latter, apparently at the cost of men's empowerment). Gender parity deteriorated significantly in households that lost access to credit in JP RWEE.

The qualitative work further shows how instrumental, intrinsic, and collective agency are interlinked for many women. Freedom of movement, work balance, and intrahousehold respect are all important so that women can participate in groups (e.g., Heifer, see Nepā School of Social Sciences and Humanities, 2017), while fear of IPV constrains women from participating (e.g., in Maisha Bora, see Krause et al., 2018). Participation in microfinance groups provides access to credit and enables women to contribute to household income and gives them confidence to speak in public (a form of intrinsic agency not captured in the index). Nor is this only at the individual level: women in the Grameen program mentioned their role in their savings group as contributing substantially to both changing norms regarding women's ability to contribute to household income as well as their own empowerment (Kieran et al., 2018).

Norm change is a transformative process that may require years to yield a measurable difference (Bicchieri and Mercier, 2014). The qualitative findings provide insight into the processes through which these changes may happen. Qualitative studies from Heifer, Grameen, SELEVER, WorldVeg, WINGS, and JP RWEE found that gender norms constrain women from participating in decisions about agricultural production overall. For instance, in Burkina Faso, focus groups showed that while women can feed or vaccinate chickens, men are considered the lead decision-makers around poultry slaughtering and marketing, and women cannot slaughter or sell chickens without their husbands' consent. It is unclear how SELEVER could affect these attitudes in the long term. In some WINGS households, spousal expectations about how women should spend their time limited their participation in groups. However, participants in three projects (ANGeL, FAARM, and Heifer) noted some norm changes, resulting in more egalitarian relations between spouses, which they attributed to project activities. FAARM found that participants reported a sustained change in decision making among their household members (Dupuis et al., 2022). But the fact that normative changes were also reported in Heifer, which did not have an explicit gender sensitization strategy despite its emphasis on women's groups, indicates that normative change may occur because of other strategies, such as forming or strengthening groups. The mixed qualitative results align with the general knowledge that norm change is not straightforward, even for projects that have an explicit strategy to address gender norms.

## 5 Discussion and conclusion

Pro-WEAI was developed as a mixed-methods approach to assess women's empowerment for agricultural development projects to diagnose^17^ key areas of women's (and men's) disempowerment, design appropriate strategies to address deficiencies, and monitor project outcomes related to women's empowerment.

### 5.1 Empowerment outcomes

Reflecting our focus on agency, we analyzed impacts on pro-WEAI's component indicators. First, we find very few significant impacts on intrinsic agency indicators, with a few exceptions from the projects that adopted intentional approaches to addressing gender norms, such as ANGeL and FAARM in Bangladesh and WorldVeg in Mali. In contrast, the qualitative findings reveal that many women report increased self-confidence based on the project interventions. The “stickiness” of the quantitative intrinsic agency indicators suggests that these may be harder to move in the short-term using current strategies, and standardized indicators may not capture the range or specific forms of intrinsic agency benefits that women themselves identify in the qualitative studies. In contrast, we find significant impacts on collective agency indicators, reflecting the group-based approaches that most projects used. Finally, we also find many significant impacts on instrumental agency indicators, probably because projects targeted such objectives as increased income or improved household nutrition.

Thus, the *intentionality* of projects to empower women is critical. Even if projects state that they have women's empowerment objectives—the vast majority of the projects in the GAAP2 portfolio do—the large number of insignificant impact estimates highlights the needs for projects to focus explicitly on empowerment, rather than assume that projects aiming to reach and benefit women would automatically empower them. The two cases with negative aggregate impacts (AVC trainings plus promotions arm, which had minimal gender content (weakly significant at p < 0.10), and JP RWEE beneficiaries who lost credit access) underscore the importance of conscious strategies and project sustainability, even to ensure that projects “do no harm” to women's empowerment. In addition, the WINGS qualitative study (Nichols 2021) identified how prior relationships between project implementers and their beneficiaries can build trust and strengthen participation and benefits over time.

Pro-WEAI is an aggregate index whose components may move in opposite directions, reflecting tradeoffs in empowerment. Because many impacts on component indicators were either null or moved in opposite directions, projects' impacts on aggregate indicators of women's empowerment (the continuous empowerment score and the binary indicator whether the woman was empowered) and gender equality (the intrahousehold inequality score) were mixed, and mostly insignificant. It is much easier for projects to impact individual components of empowerment in the short term than to significantly change the aggregate indicators.

We also find consistent differences in the patterns across regions, possibly because these regions have different types of “patriarchal bargains” (Kandiyoti 1988). Projects in South Asia were more likely to show significant impacts on women's empowerment than those in Africa, perhaps reflecting a longer history and more experience with designing programs to address particular forms of women's disempowerment found in the patrilocal extended household characteristic of “classic patriarchy” in South and East Asia (Kandiyoti 1988, p. 278). The negative impacts on men's aggregate indicators in some Africa projects may because for concern, if these create potential for backlash. Some of the Africa projects may have been designed without adequately considering the prevalent type of patriarchy in the region, where women may have relative autonomy over specific domains (Kandiyoti 1988) although this varies greatly across sub-Saharan Africa. The variability in project impacts on empowerment both within and across regions is consistent with the variability Lombardini and McCollum (2018) found across Oxfam projects, although they found a higher proportion of projects having overall positive impacts. Because family structures differ greatly across contexts, designing interventions for men and women that consider their social position relative to other household members is key to successfully changing gender norms.

Our findings reinforce the need to pay attention to both project implementation and context. The mixed results of projects on tolerance of IPV illustrate the importance of both. The three projects (ANGeL, FAARM, and WorldVeg) where beneficiaries reported an increase in the number of instances for which respondents said that IPV was not justified indicate a heightened critical consciousness of what is (and is not) acceptable in spousal relationships. In the other projects where women identify fewer instances in which IPV is unjustified, it may indicate that women are willing to tolerate more instances of IPV in exchange for other types of freedoms. Qualitative findings from the Grameen project found that empowered women are perceived to be “autonomous” yet “submissive” to their husbands and families (Kieran et al., 2018). This is similar to reports from another project among the Afar in Ethiopia that women gain social status by submitting to IPV without protest, and that increase in status is associated with empowerment (Mosedale 2014:1121).

While our small sample size of 11 projects and 32 treatments prevented us from conducting a robust quantitative analysis relating strategies adopted by projects with the direction and magnitude of the estimated impacts, a qualitative assessment of these patterns provides important insights. Although many projects adopted similar strategies, there did not seem to be a single effective strategy that worked across contexts. Instead, it may be more important that the strategy be adapted to local needs and implemented well. We recommend that projects that seek to empower women pay more attention to ensuring that they have strategies that go beyond reaching and benefitting women and think critically about what activities would contribute to different types of empowerment. Such intentionality goes beyond stating that a project has empowerment objectives; it involves having strategies that work to empower women in their specific contexts. A synthesis of impact evaluations across four countries in the UN JP RWEE portfolio (Ethiopia, Kyrgyzstan, Nepal, and Niger; the Ethiopia study is part of GAAP2) found positive impacts that are significant and larger in magnitude on women's empowerment and gender equality (Quisumbing et al., 2023) compared to the projects in our portfolio. The JP RWEE synthesis study attributes this to having approaches that explicitly target gender norms and work with men.

Because the projects were implemented in different contexts, we can consider patterns and linkages across different dimensions of empowerment. The qualitative studies provide nuance and insight into how projects affected women's empowerment and linkages among the different types of agencies (Meinzen-Dick et al., 2019). For example, freedom of movement and work balance (instrumental agency) and respect among family members (intrinsic agency) may be needed for women to be able to join groups (collective agency); group membership, in turn, is reported to increase access to credit, control over income, and input into productive decisions (instrumental agency) as well as women's self-confidence (intrinsic agency). Thus, some base level and forms of agency may be necessary for women to be able to participate in project activities, to benefit or to increase their empowerment. Identifying these linkages and baseline information about each aspect of empowerment can help projects to adapt their strategies, such as ensuring that women have freedom of movement if they are expected to attend group meetings or training.

Moreover, programs may need to provide sustained exposure to the intervention to maximize the potential for projects to benefit and empower women. Those that are not sufficiently intensive in their approaches, such as community-based programs with selective uptake of multiple project components, may not provide sufficient exposure and have more limited empowerment outcomes, such as may have been the case with SELEVER (Heckert et al., 2023). Some base level, not only of empowerment, but more importantly of resources needed to take up interventions (time, material, and financial resources) may also be necessary for projects to succeed. Findings across relatively “light-touch” projects, such as WINGS and SELEVER, suggested that in exceptionally poor contexts, women and their households may also need a baseline level of resources or potentially asset transfers to be able to benefit from or be empowered by agricultural development projects. This is particularly true in livelihood-focused projects that require significant capital investments.

We note that empowerment is also an ongoing and iterative process, in which each stage in the process contributes to further empowerment; if this process is interrupted, then women may have difficulty further empowering themselves (Dupuis et al., 2022). The negative outcomes for women who lost credit access in the Ethiopia JP RWEE project provides a cautionary note in this regard.

### 5.2 The importance of mixed methods

Our efforts illustrate the importance of complementing quantitative impact evaluations with qualitative investigations and process evaluations. The development of pro-WEAI started with WEAI indicators, which were informed by qualitative life histories (Alkire et al., 2013) and GAAP2 has used qualitative and quantitative methods since its inception. Qualitative methods (review of project documents) were used to identify the strategies that projects used to empower women and to inform the design of quantitative modules that were included in pro-WEAI. Partner projects participated in choosing the indicators to be tested in the pilot version of pro-WEAI; these indicators were then validated using the qualitative protocols that are part of the standard pro-WEAI toolkit and underwent further assessment using psychometric methods (Yount et al., 2019).

Qualitative methods were also used to “ground truth” the findings on the meaning of empowerment as well as the sources of disempowerment (Meinzen-Dick et al., 2019). Qualitative methods helped us to understand beneficiaries’ experiences of empowerment (or lack thereof) associated with the projects and can be used to contextualize and explain quantitative findings, such as whether data is from busy or slack seasons. Additionally, combining qualitative and quantitative methods allowed us to probe each set of results more deeply. This ability was particularly important when the qualitative and quantitative methods found differing results. In such instances, using mixed methods helped to illuminate issues that would not have been exposed by either method individually, ultimately allowing us to develop more comprehensive and salient indicators. Without the nuance provided by the qualitative results, we could have erred on the side of coming up with quantitative indicators that did not measure anything meaningful, as Tavenner and Crane (2022) have cautioned against.

We are also mindful of the limitations of qualitative data: the limited number, both of respondents and of the select beneficiary communities where qualitative studies were conducted, does not allow us to assess how widespread the changes are. Although we tried to include diverse respondents, it is possible that the more articulate, and empowered, respondents had greater voice.

The pro-WEAI suite of methods allows us to have the best of both worlds: a quantitative, standardized instrument that is comparable across a project portfolio, and qualitative protocols that provide insights into the local, context-specific meanings of empowerment and the processes underlying empowerment (or disempowerment) associated with agricultural development projects. While the aggregate pro-WEAI score and proportion of women (and men) who are empowered or disempowered are useful for diagnosing disempowerment, they may not provide enough information for impact evaluations to assess whether the intervention is working. Although the individual indicators provide more detail on how interventions may affect empowerment, the pro-WEAI indicators may be too coarse to pick up some project impacts. For example, the continuous indicator for group membership, defined as the number of types of groups to which a respondent belongs, will capture improvements that lead to membership in new types of groups but will not reflect improvements in the quality of the member's participation in an existing group. The richness of the qualitative insights on collective agency suggests that developing better quantitative indicators of collective agency will be an important area for future research. A similar argument could be made for indicators of input in productive decisions and control over use of income, defined as numbers of types of activities in which a respondent demonstrates agency. Even the so-called “continuous” indicator of assets refers to the number of asset categories, rather than the value of assets. Women might be acquiring fewer types of assets, but more valuable ones and this would not be captured. Various trade-offs made in choosing the indicators may not capture every nuance of interest.

### 5.3 Recommendations for use

For metrics like pro-WEAI that aim to help projects monitor progress toward their empowerment objectives, we have both recommendations and words of caution. First, use both qualitative and quantitative tools and methods. This process begins with a review of project documents to identify the project's theory of change and impact pathways linking strategies to empowerment. Other qualitative instruments provide an important understanding of how project staff as well as local women and men view women's empowerment, and how the project may (or may not) be contributing. Turning to the quantitative data, collecting survey data from both men and women is necessary to measure gender equality, and to identify whether women's disempowerment is primarily gender-based, or whether men in their households are also disempowered. The 3DE and GPI, as composite pro-WEAI indicators, are useful for characterizing overall changes in empowerment, but changes in individual indicators can better identify where a project is having greatest (or least) success. Analysis of the individual indicators can also identify possible trade-offs, such as increased workloads accompanying women's increased participation in decision making.

In this regard, pro-WEAI can be useful as a diagnostic, particularly if implemented early in the project cycle, to assess which aspects of agency are most important to address for women and for men. We caution project designers and implementors against setting targets based on specific levels of change in pro-WEAI or its indicators. We do not yet have enough evidence to guide decisions on what levels of change are meaningful for different project settings. Our findings suggest that pro-WEAI can detect impacts on most aspects of agency that can change over the course of a typical project timeline (for example, instrumental agency), but may not be capable of detecting impacts on aspects of agency, such as intrinsic agency, which are slower to change because of underlying norms and gender attitudes (Bicchieri and Mercier, 2014). Qualitative work may be better able to capture subtle changes related to norms and attitudes, such as local meanings of empowerment (O'Hara and Clement 2018). Thus, we strongly recommend that qualitative work be conducted in tandem with quantitative evaluations.

Because of the project- and context-specificity of impact evaluation results, we cannot identify “best practices” or “proven strategies”; indeed, as Johnson (2021) notes, it may be advisable to replace these phrases with more nuanced language and move toward “approaches” that support the design of more effective projects. Each project needs to use a solid diagnosis of gender relations and women's constraints to develop strategies that are appropriate to that context. With more consistent characterization and analysis of strategies, it may be possible for future GAAP2–like projects to identify patterns in how specific strategies work in different contexts to provide broader guidance on how they could be implemented or adapted.

Finally, it is important to recognize that pro-WEAI measures empowerment, but impact assessments should be designed with appropriate counterfactuals or control groups and collect data on other outcomes, such as increased productivity, incomes, nutrition, or environmental conditions. These can map to the other key aspects of empowerment: resources and achievements (Kabeer 1999). Such data are important not only for the projects themselves to assess their success, but also to build the evidence base on the association between women's empowerment and other development objectives.

### 5.4 Conclusion

Can agricultural development projects increase women's agency and improve empowerment outcomes? Our answer, based on qualitative and quantitative impact evaluations across a portfolio of projects, is a qualified “yes.” Although we did not find a single “best strategy” that always led to positive outcomes, projects in our portfolio that improved women's agency and empowerment outcomes overall or particular indicators were intentional about their project strategies, had activities adapted to culture and context, and paid attention to unintended consequences (notably backlash from men or increased workload).

The projects in our portfolio were developed without a clear sense of differences between “reach”, “benefit”, “empower” and “transform” or the specific aspects of empowerment to which they might contribute. The advances in measurement of empowerment and conceptualization of how projects can influence empowerment since then can help future projects be more intentional and successful. Using qualitative tools to understand context and baseline pro-WEAI results as a diagnostic, future projects can identify the most appropriate empowerment strategies for their particular context. For example, the high levels of disempowerment on the IPV indicator at baseline in the Maisha Bora case and the qualitative information on how IPV restricted women's participation in groups and markets, hence control over income, prompted the implementing organization to add an IPV reduction component to a new project. Understanding from the qualitative studies also provides insights on how different dimensions of empowerment are linked can also lead to more effective programming that addresses multiple constraints (e.g. household support, mobility, group membership, credit, control over income, and intrinsic agency). Such insights can inform project strategies that go beyond “reach” to “benefit” and even “empower”.

## CRediT authorship contribution statement

**Agnes R. Quisumbing:** Conceptualization, Methodology, Investigation, Writing – original draft, Writing – review & editing, Supervision, Project administration, Funding acquisition. **Ruth Meinzen-Dick:** Conceptualization, Methodology, Investigation, Writing – original draft, Writing – review & editing, Supervision, Project administration, Funding acquisition. **Hazel J. Malapit:** Conceptualization, Methodology, Validation, Investigation, Writing – original draft, Writing – review & editing, Supervision, Project administration, Funding acquisition. **Greg Seymour:** Conceptualization, Methodology, Validation, Investigation, Writing – original draft, Writing – review & editing. **Jessica Heckert:** Conceptualization, Methodology, Validation, Investigation, Writing – original draft, Writing – review & editing. **Cheryl Doss:** Conceptualization, Methodology, Investigation, Writing – original draft, Writing – review & editing. **Nancy Johnson:** Conceptualization, Methodology, Investigation, Writing – original draft. **Deborah Rubin:** Conceptualization, Methodology, Investigation, Writing – original draft, Writing – review & editing. **Giang Thai:** Methodology, Software, Validation, Formal analysis, Investigation, Visualization. **Gayathri Ramani:** Methodology, Software, Validation, Formal analysis, Investigation, Visualization. **Emily Myers:** Methodology, Formal analysis, Validation, Investigation, Writing – original draft, Writing – review & editing. **GAAP2 for pro-WEAI Study Team:** Conceptualization, Methodology, Validation, Formal Analysis, Investigation, Resources, Data Curation, Writing – original draft, Writing – review & editing.

## Declaration of competing interest

The authors declare no competing interests.

## Figures and Tables

**Appendix Table 1:** Projects in the GAAP2 portfolio

Project acronym	Project name	Implementer and evaluator	Project modality/goal	Project objective	Project approach
**South Asia/Bangladesh**					
**ANGeL**	Agriculture, Nutrition, and Gender Linkages	Ministry of Agriculture (Bangladesh) and International Food Policy Research Institute (IFPRI)	Crops/Nutrition	To pilot alternative approaches to integrating agriculture, nutrition, and women's empowerment, the most effective of which will be scaled up.	Provide training on three approaches in different combinations: facilitating production of nutrient-rich food, conducting high-quality behavior change communication (BCC), and undertaking gender sensitization activities. All trainings delivered to husbands and wives jointly
**AVC**	Impact Evaluation of the Bangladesh Agricultural Value Chains Program	Development Alternatives International (DAI) and IFPRI	Crops/Income and nutrition	To increase agricultural output and income, and improve food and nutrition security through strengthened agricultural value chains	Conduct trainings to build farmers' capacity in using improved seed varieties and cultivation practices along with basic training on gender and nutrition issues and provision of promotional discounts on fertilizer and seeds to incentivize adoption
**FAARM**	Food and Agricultural Approaches to Reducing Malnutrition	Helen Keller International (HKI) and University of Heidelberg	Crops and livestock/Nutrition	To reduce undernutrition among women and young children through a food-based dietary diversification strategy and to increase the status of women within the household	Intervention based on HKI's enhanced homestead food production model involves training rural women's groups in vegetable gardening, fruit tree production, and poultry rearing, along with nutrition and hygiene
**TRAIN**	Targeting and Realigning Agriculture to Improve Nutrition	BRAC and IFPRI	Crops and livestock/Nutrition	To increase the dietary diversity of poor rural producers by 1) increasing the diversity of crops grown and income generated; 2) improving child feeding, health, and sanitation practices using BCC; and 3) empowering women by facilitating greater control over agricultural income and its allocation toward health and nutrition as well as sensitizing husbands to support wives in productive and reproductive tasks.	Using a randomized controlled trial, assess the impact of incorporating agricultural interventions to promote production, production diversity and income-generation into a strong state-of-the-art maternal and child health and nutrition BCC platform
**South Asia/India**					
**WINGS**	Women Improving Nutrition through Group-Based Strategies	Professional Assistance for Development Action (PRADAN), Public Health Resource Society (PHRS), and IFPRI	Crops and livestock/Nutrition	To improve women's and children's diets and nutrition outcomes through increasing own consumption and income Existing SHG platform has women's empowerment objectives	Uses existing women's self-help groups to deliver BCC and training on nutrition-sensitive agricultural planning, and works with the community and public systems/institutions to ensure that services of public health and nutrition programs are available and accessible in the project area
**South Asia/Nepal**					
**Heifer**	Empowerment, Resilience, and Livestock Transfers	Heifer International and Montana State University	Livestock/Income and nutrition	To increase income, food security and nutrition, and women's empowerment, and improve aspirations, hope, and economic resilience among the chronically poor by building physical, human, and social capital	Provides women with livestock transfers and training related to nutrition, home gardening, and livestock management; forms self-help groups through which women receive empowerment training
**West Africa/Burkina Faso**					
**Grameen Foundation**	Building Resilience of Vulnerable Communities in Burkina Faso	Grameen Foundation and Brigham Young University	Crops and livestock/Income and nutrition	To increase the resilience of vulnerable communities in disaster-affected regions by building women's economic empowerment, and to strengthen women's capacity to make decisions about children's nutrition	Uses community-based women's savings groups as a sustainable platform for improving livelihoods through training, education on agriculture as a business, linkages to agricultural services, financing for common agricultural activities, nutrition education, and gender dialogues
**SELEVER**	Soutenir l’Exploitation Familiale pour Lancer l’Elevage des Volailles et Valoriser l’Economie Rural (Women's Poultry Program to Improve Income and Nutrition)	Agribusiness Systems International, AfricSanté, and IFPRI	Livestock/Nutrition and income	To increase poultry production and improve the nutritional status of women and children in the Centre-Ouest, Hauts-Bassins and Boucle de Mouhoun regions of Burkina Faso	Uses an integrated market-facilitation approach combining revenue generation, women's empowerment, and nutritional behavior change interventions
**West Africa/Ghana**					
**iDE**	Small-Scale Irrigation and Women's Empowerment in Northern Ghana	iDE and IFPRI	Crops/Income and nutrition	To expand production of food during the lean season and reduce production risks during rainy seasons through small-scale irrigation, which will increase income, food security, nutrition, and health	Provides women access to motor pumps along with training, access to credit, and other agricultural inputs
**West Africa/Mali**					
**WorldVeg**	Integrated home garden project	World Vegetable Center	Crops/Income and nutrition	To improve nutritional status and dietary diversity by increasing vegetable production and consumption	Integrated home garden project—combining training in gardening with nutrition behavior change communication and training in water, sanitation, and hygiene (WASH)
**East Africa/Ethiopia**					
**JP-RWEE**	UN Joint Programme on Accelerating Progress towards the Economic Empowerment of Rural Women in Ethiopia	Food and Agriculture Organization of the United Nations (FAO) and International Fund for Agricultural Development (IFAD)	Crops and livestock/Income and nutrition	To reduce gender inequalities in pastoralist communities related to access to resources, credit, and financial services to improve household food security, women's decision making within the household, and women's participation in the community	Interventions include strengthening associations and cooperatives to offer financial products to women farmers, providing credit to women farmers, and giving women financial literacy and entrepreneurship training
**East Africa/Kenya**					
**MoreMilk**	MoreMilk: Making the most of milk	International Livestock Research Institute	Livestock/Income and nutrition	To enhance milk safety and child nutrition in peri-urban Nairobi	Training milk traders to improve their milk handling and business practices
East Africa/Tanzania					
**Maisha Bora**	Evaluation of Women's Food Security Program for Impoverished Maasai Households	Savannas Forever and University of Minnesota	Livestock/Income and nutrition	To increase food security of semi pastoralist communities through a more diversified and secure income from improvements in livestock	Builds capacity of pastoralists' organizations to provide entrepreneurship training, business skills training, and advocacy for women; forms savings and credit groups and women-only farms; provides training on household budgeting and gender awareness

**Appendix Table 2:** Project evaluation design, empowerment metrics, and qualitative work^1^.

Project acronym	Country	Quantitative evaluation design	Sample selection and sample size for pro-WEAI analysis	Survey dates	WEAI metric collected	Qualitative tools	Dates of qualitative data collection
**South Asia**							
**ANGeL**	Bangladesh	Randomized controlled trial	Farming households with at least one child younger than 24 months Women: 2739 Men: 2739	Baseline: Nov 2015–Jan 2016 Endline: Jan–March 2018	Baseline: A-WEAI Endline: pro-WEAI	FGDs with beneficiaries IDIs with beneficiaries	April to June 2018 (break for Eid)
**AVC**	Bangladesh	Randomized controlled trial	Households intending to plant jute and cultivating at most 2.02 ha Women: 477 Men: 457	Baseline: February–March 2016) Midline: February–March 2017 Endline: April–May 2018	Baseline: A-WEAI Midline and endline: pro-WEAI	FGDs with beneficiaries KIIs with Agricultural officers. input dealers, and traders	January to February 2018
**FAARM**	Bangladesh	Randomized controlled trial	Married women aged 15–30 years Women: 457 Men: 449	Baseline: March to May 2015 Pro-WEAI data collection: April to May 2019	Endline: pro-WEAI	FGDs with beneficiaries IDIs with beneficiaries and non-beneficiaries KIIs with market traders, program staff, community leaders, group leaders Life history interviews with beneficiaries Seasonal calendars	September to October 2017 February 2018 February 2019 June to July 2019
**WINGS**	India	Inverse probability weighted difference-in-difference	Households with at least one ever-married female member between 15 and 49 years old Women: 1333 Men: 1330	Baseline: Nov 2015–Jan 2016 Midline: November 2017 to January 2018 Endline: November 2019–January 2020	Baseline: A-WEAI Midline and endline: pro-WEAI	FGDs with program beneficiaries IDIs with program beneficiaries KIIs with project staff	June 2019
**Heifer**	Nepal	Randomized controlled trial	Women eligible to participate in Heifer program Women: 1817 Men: empowerment data were not collected	Baseline: Mid 2014 Endline: Mid 2016	Baseline and endline: A-WEAI	FGDs IDIs with beneficiaries and non-beneficiaries Life history interviews with women and men Observation Seasonal calendar	March to April 2017
**West Africa**							
**FFH/Grameen Foundation**	Burkina Faso	Difference-in-differences	Women who were members of savings groups Women: 380 Men: 380	Baseline: May 2016 Endline: November 2017	Baseline and endline: pro-WEAI	Community profile FGDs with beneficiaries KIIs with project staff and market traders Life history interviews with beneficiaries Seasonal calendar	Baseline: October–November 2015 Endline: January 2019
**SELEVER**	Burkina Faso	Randomized controlled trial	Households with women aged 15–35 years and at least one child aged 2–4 years Women: 1487 Men: 1396	Baseline: March 2017 (post-harvest) Lean season surveys: September 2017 September 2019 Endline: March 2020, postponed, resumed in August	Baseline and endline: pro-WEAI	FGDs with beneficiaries and community members IDIs with beneficiaries, poultry producers, poultry traders, village vaccinators, group leaders Seasonal calendars	January to March 2019
**iDE**	Ghana	Treatment villages randomly selected from stratified pairs; trust groups within treatment villages selected by lottery	Households in three groups: 1) lottery winners in early treatment villages, 2) lottery losers in early treatment villages (and non-participants), and 3) farmers who formed groups in control villages that did not participate in the lottery. Women: 747 Men: empowerment data not collected	Baseline: November 2015 to early February 2016 Endline: December 2017 and February 2018	Baseline: A-WEAI and endline: pro-WEAI	FGDs with beneficiaries and non-beneficiaries KIIs with market traders Life history interviews with beneficiaries and non-beneficiaries Seasonal calendar	July to August 2017
**WorldVeg**	Mali	Difference-in-difference with entropy balancing	Households with women aged 15–49 years and oversampled households with a child younger than five years old Women: 560 Men: 560	Baseline: September to October 2016 Endline: September to October 2018	Baseline and endline: pro-WEAI	Community profile FGDs with beneficiaries and non-beneficiaries KIIs with project staff and market traders Life history interviews with beneficiaries and non-beneficiaries Seasonal calendar	December 2016 and January 2017
**East Africa**							
**JP-RWEE**	Ethiopia	Difference-in-difference with Inverse Probability Weighting	Members of RUSSACO in beneficiary communities at baseline; comparison group randomly drawn from two comparable kebeles in three woredas All women: 723 Women in couple households: 528 Men in couple households: 528	Baseline: December 2016 to January 2017 Midline: February to March 2019	Baseline and midline: pro-WEAI	FGDs with beneficiaries and spouses of beneficiaries IDIs with beneficiaries and spouses of beneficiaries KIIs with project implementers and kebele leaders	July to August 2017
**Maisha Bora**	Tanzania	Propensity weighted difference-in-difference	Households randomly drawn from sub-village registers, 60 households from 14 villages, half of which received the Maisha Bora program treatment Women: 681 (both baseline and endline) Men: 154 (both baseline and endline)^2^	Baseline: December 2015–May 2016 Endline: December 2019–February 2020	Baseline and endline: pro-WEAI	FGDs with beneficiaries IDIs with beneficiaries KIIs with project staff; village leaders	December 2016 (KIIs with project staff) June 2017 (all others)

^2^Sample size for pro-WEAI impact analysis may be smaller than the sample for the impact evaluation where pro-WEAI was collected for a subsample of households. Numbers of women and men reported are from the combined control and intervention samples.

1 This refers to the 11 GAAP2 projects that completed their impact evaluations in 2020.

2 Maisha Bora interviewed 225 men at baseline, but owing to the challenges of interviewing pastoralists, interviewed only 154 at both baseline and endline. They did not analyze men's outcomes owing to the small sample size.

**Appendix Table 3:** Pro-WEAI qualitative protocols

Protocol	Method and purpose
Review of project documents	• Method: Desk review completed by member of research team • Purpose: Examine the study context, a project's theory of change, and the different components of the intervention evaluated
Community profile	• Method: Group activity with 2–3 community members in a few different locations in the study area • Purpose: Understand migration patterns, infrastructure, information communication technologies, education, natural resources, healthcare, and marriage practices in the study context
Seasonality calendar	• Method: Group activity with 4–5 women and men • Purpose: Develop a production calendar showing agricultural activities distributed by gender and age over the course of a year to see how seasonal variations affect time use
Local understandings of empowerment	• Method: Sex-disaggregated focus groups of women and men • Purpose: Understand the various ways empowerment is perceived among the study population
Life histories	• Method: Semi-structured interviews with women and men • Purpose: Gather information about key events and experience in respondents' lives, related to agriculture and other topics to uncover patterns across individuals and groups
Key informant interviews with market traders	• Method: Semi-structured interviews with market traders, both women and men • Purpose: Examine how women and participate in different nodes in relevant value chains, such as engaging with formal and informal markets, the assets needed to work in a particular node, and gendered constraints to participating in particular nodes
Key informant interviews with project staff	• Method: Semi-structured interviews with project staff • Purpose: Elicit insights into how the project affects women's empowerment, whether staff are knowledgeable about the project's empowerment objectives, and whether they support those objectives to enhancing project performance, learning about the viability of bringing projects to scale, and ultimately achieving gender equality.
Additional questions specific to project strategies	• Awareness and benefits of the project activities such as trainings and/or provision of inputs (e.g., credit, seeds, livestock, agricultural equipment) • Project participants' perspectives on gender sensitization strategies and/or their group participation • Relationships between project participants and project staff.

**Appendix Table 4:** GAAP2 portfolio and sample size of the qualitative research component

Project name	Country	Key informant or life history interviews			Focus groups (N)
		Total (N)	Women (N)	Men (N)	
**South Asia**					
ANGeL^1^	Bangladesh	26 (13)	14 (8)	12 (5)	5 (2)
AVC	Bangladesh	54	17	37	16
FAARM	Bangladesh	44	22	22	12
WINGS	India	65	58	7	6
Heifer^2^	Nepal	34	N/A	N/A	21
**Africa**					
Grameen	Burkina Faso	14	6	8	4
SELEVER	Burkina Faso	53	24	29	24
JP RWEE	Ethiopia	68	30	38	40
iDE	Ghana	24	16	8	12
WorldVeg	Mali	19	16	3	12
Maisha Bora	Tanzania	52	26	26	14

Notes: See Appendix Table 1 for the full names of projects and implementing organizations.

1 Numbers outside parentheses refer to interviews conducted among project beneficiaries; those in parentheses refer to interviews or focus groups conducted specifically for the empowerment analysis.

2 The gender identity of participants was not reported. N/A: not available.

**Appendix Table 5:** Comparing Pro-WEAI and A-WEAI domains and indicators

Pro-WEAI domain	Pro-WEAI indicator name	Pro-WEAI definition	A-WEAI domain	A-WEAI indicator name	A-WEAI definition
Intrinsic agency	Autonomy in income	More motivated by own values than by coercion or fear of others' disapproval *Weight = 1/12*	–	–	–
Intrinsic agency	Self-efficacy	“Agree” or greater on average with self-efficacy questions: *New General Self-Efficacy Scale*^C^ score ≥ 32 *Weight = 1/12*	–	–	–
Intrinsic agency	Attitudes about intimate partner violence against women	Believes husband is NOT justified in hitting or beating his wife in all 5 scenarios:^D^ 1) She goes out without telling him 2) She neglects the children 3) She argues with him 4) She refuses to have sex with him 5) She burns the food *Weight = 1/12*	–	–	–
Intrinsic agency	Respect among household members	Meets ALL the following conditions related to their spouse, the other respondent, or another household member: 1) Respondent respects relation (MOST of the time) AND 2) Relation respects respondent (MOST of the time) AND 3) Respondent trusts relation (MOST of the time) AND 4) Respondent is comfortable disagreeing with relation (MOST of the time) *Weight = 1/12*	–	–	–
Instrumental Agency	Input in productive decisions	Meets at least ONE of the following conditions for ALL the agricultural activities they participate in 1) Makes related decision solely, 2) Makes the decision jointly and has at least some input into the decisions 3) Feels could make decision if wanted to (to at least a MEDIUM extent) *Weight = 1/12*	Production	Input in productive decisions	Adequate if individual participates in and makes decisions, has input in decisions, or feels she could make decisions (if desired) about at one agricultural activity *Weight = 1/5*
Instrumental Agency	Ownership of land and other assets	Owns, either solely or jointly, at least ONE of the following: (updated March 2020) 1) Any three assets 2) Land *Weight = 1/12*	Resources	Ownership of assets	Adequate if individual owns at least one major asset or at least two minor assets *Weight = 2/15*
Instrumental Agency	Access to and decisions on financial services	Meets at least ONE of the following conditions: 1) Belongs to a household that used a source of credit in the past year AND participated in at least ONE sole or joint decision about it 2) Belongs to a household that did not use credit in the past year but could have if wanted to from at least ONE source 3) Has access, solely or jointly, to a financial account *Weight = 1/12*	Resources	Access to and decisions about credit	Adequate if individual makes decisions about at least one source of credit accessed by her/his household *Weight = 1/15*
Instrumental Agency	Control over use of income	Has input in decisions related to how to use BOTH income and output from ALL the agricultural activities they participate in AND has input in decisions related to income from ALL non-agricultural activities they participate in, unless no decision was made *Weight = 1/12*	Income	Control over use of income	Adequate if individual participates in and has input in decisions about income generated from an activity or she/he makes decisions, has input in decisions, or feels she/he could make decisions (if desired) about employment or major household expenditures *Weight = 1/5*
Instrumental Agency	Work balance	Works less than 10.5 h per day: Workload = time spent in primary activity + (1/2) time spent in childcare as a secondary activity *Weight = 1/12*	Time	Workload	Adequate if individual worked fewer than 10.5 h during the previous day *Weight = 1/5*
Instrumental Agency	Visiting important locations	Meets at least ONE of the following conditions: 1) Visits at least TWO locations at least ONCE PER WEEK of [city, market, family/relative], or 2) Visits least ONE location at least ONCE PER MONTH of [health facility, public meeting] *Weight = 1/12*	–	–	–
Collective Agency	Group membership	Active member of at least ONE group *Weight = 1/12*	Leadership	Group member	Adequate if individual is an active member of at least one group *Weight = 1/5*
Collective Agency	Membership in influential groups	Active member of at least ONE group that can influence the community to at least a MEDIUM extent *Weight = 1/12*	–	–	–

**Appendix Table 6:** Project impacts on women's intrinsic agency and collective agency indicators (continuous)

	Intrinsic agency								Collective agency			
	Relative autonomy index score (range: 3 to −3)		Self-efficacy scale score (range: 8 to 40)		Number of situations in which intimate partner violence is not justified		Number of conditions met defining respect		Number of types of groups to which respondent belongs		Number of types of groups regarded as influential to which respondent belongs	
	Endline control mean	Impact	Endline control mean	Impact	Endline control mean	Impact	Endline control mean	Impact	Endline control mean	Impact	Endline control mean	Impact
**South Asia**												
***ANGeL***^1^, ^2^												
Control	1.67 (1.81)		30.09 (5.64)		4.09 (1.41)		3.54 (0.84)		0.52 (0.64)		0.24 (0.51)	
T-N		0.05 (0.04)		0.06 (0.05)		0.03 (0.04)		−0.03 (0.04)		0.04 (0.04)		0.02 (0.04)
T-A		0.01 (0.04)		0.08∗ (0.04)		0.04 (0.04)		0.01 (0.04)		0.00 (0.04)		−0.02 (0.04)
T-AN		0.00 (0.04)		0.04 (0.05)		0.01 (0.04)		0.02 (0.04)		0.02 (0.03)		0.03 (0.04)
T-ANG		0.08∗ (0.04)		0.02 (0.05)		0.08∗ (0.04)		0.02 (0.04)		0.09∗∗ (0.04)		0.04 (0.04)
***AVC***^2^^,^^3^												
Control	1.43 (2.06)		31.95 (4.04)		4.27 (1.16)		3.39 (0.83)		0.43 (0.58)		0.13 (0.34)	
NGO trainings only		−0.05 (0.56)		−0.05 (0.56)		−0.05 (0.19)		−0.10 (0.15)		0.06 (0.09)		0.11 (0.05)
NAAFCO promotions only		0.10 (0.37)		−0.05 (0.50)		0.01 (0.15)		−0.02 (0.17)		−0.08 (0.09)		−0.07 (0.04)
Trainings + promotions		0.00 (0.35)		0.13 (0.48)		0.08 (0.17)		−0.20 (0.15)		−0.10 (0.08)		−0.06 (0.05)
***FAARM***^2^												
Control	1.31 (1.84)		27.92 (4.57)		4.43 (1.05)		1.14 (1.29)		0.33 (0.52)		0.00 (0.10)	
Intervention		0.11∗∗∗ (0.04)		0.30∗∗∗ (0.04)		0.19∗∗∗ (0.04)		0.06 (0.05)		0.40∗∗∗ (0.04)		0.44∗∗∗ (0.05)
***WINGS***^4^												
Control	−0.12 (1.82)		16.41 (16.07)		4.54 (1.04)		3.43 (0.79)		0.37 (0.53)		0.17 (0.39)	
NI (DD)		n.c.		n.c.		−0.11∗∗ (0.04)		0.09∗ (0.05)		−0.02 (0.04)		0.01 (0.05)
NI (SD)		0.04 (0.04)		−0.08∗∗ (0.04)		−0.15∗∗∗ (0.04)		−0.02 (0.04)		0.10∗∗∗ (0.04)		0.06 (0.04)
***Heifer (A-WEAI)***^2^, ^5^												
*Direct beneficiary*												
Control							5.95 (0.13)					
Full treatment		n.c.		n.c.		n.c.		−0.11 (0.09)				n.c.
Values-based training		n.c.		n.c.		n.c.		0.03 (0.08)				n.c.
Goats		n.c.		n.c.		n.c.		−0.03 (0.08)				n.c.
*Pay-it-forward Beneficiary*												
Control							5.74 (0.15)					
Full treatment		n.c.		n.c.		n.c.		−0.08 (0.10)				n.c.
Values-based training		n.c.		n.c.		n.c.		0.08 (0.10)				n.c.
Goats		n.c.		n.c.		n.c.		−0.09 (0.10)				n.c.
**West Africa**												
***Grameen***^6^												
Control	0.80 (2.03)				3.85 (1.52)		3.17 (1.65)		2.71 (1.39)		3.96 (0.21)	
Intervention		−0.79∗∗∗ (0.15)				−0.65∗∗∗ (0.14)		0.12 (0.15)		0.81∗∗∗ (0.14)		0.44∗∗∗ (0.14)
***SELEVER***^7^												
Control	0.20 (2.12)		30.11 (4.61)		3.66 (1.71)		3.41 (0.87)		0.61 (0.72)		0.50 (0.68)	
SELEVER		0.03 (0.04)		−0.03 (0.03)		−0.02 (0.04)		−0.03 (0.03)		−0.02 (0.03)		−0.00 (0.03)
***iDE***^6^												
Control 1									1.41 (1.11)			
Motor pump (control group 1, DD)		n.c.		n.c.		n.c.		n.c.		−0.06 (0.05)		n.c.
Control 2									1.50 (1.20)			
Motor pump (control group 2, DD)		n.c.		n.c.		n.c.		n.c.		−0.03 (0.06)		n.c.
Motor pump spillover effects, DD)		n.c.		n.c.		n.c.		n.c.	1.50 (1.20)	−0.04 (0.05)		n.c.
Control 1			27.72 (7.69)		3.63 (1.70)				1.40 (1.18)		0.76 (0.81)	
Motor pump (control group 1, SD)		n.c.		0.02 (0.05)		0.00 (0.06)		n.c.		0.07 (0.06)		0.05 (0.05)
Control 2			27.84 (7.79)		3.67 (1.66)			n.c.	1.48 (1.30)		0.84 (0.85)	
Motor pump (control group 2, SD)				−0.27 (0.17)		0.02 (0.12)				0.10 (0.15)		−0.06 (0.14)
Motor pump spillover effects, SD)			27.84 (7.79)	0.10 (0.13)	3.67 (1.66)	−0.27∗ (0.14)			1.48 (1.30)	0.16 (0.13)	0.84 (0.85)	0.01 (0.16)
***WorldVeg***^8^												
Control	−0.10 (1.66)		31.55 (3.41)		3.26 (1.77)		3.20 (1.13)		1.61 (1.38)		1.20 (1.12)	
Intervention (ITT)		0.06 (0.08)		−0.02 (0.07)		0.17∗∗ (0.08)		−0.03 (0.07)		−0.03 (0.09)		−0.07 (0.08)
Intervention (TOT)		0.07 (0.07)		0.01 (0.05)		0.01 (0.08)		−0.07 (0.07)		0.06 (0.07)		0.08 (0.06)
**East Africa**												
***JP-RWEE***^9^												
Control	1.43 (1.55)				3.96 (1.65)				1.12 (0.82)			
*All women*												
With access to credit		0.04 (0.05)		n.c.		−0.05 (0.04)		n.c.		0.07∗ (0.04)		
Lost access to credit		−0.16∗∗∗ (0.05)		n.c.		−0.12∗∗ (0.05)		n.c.		−0.16∗∗∗ (0.05)		
*Women in primary couple*												
	1.40 (1.55)				3.96 (1.64)		3.16 (1.20)		0.99 (0.81)		0.47 (0.67)	
With access to credit		0.02 (0.06)		n.c.		−0.07 (0.05)		0.05 (0.05)		0.11∗∗ (0.05)		0.10∗ (0.06)
Lost access to credit		−0.20∗∗∗ (0.06)		n.c.		−0.21∗∗∗ (0.06)		−0.04 (0.05)		−0.14∗∗∗ (0.05)		−0.10∗∗ (0.05)
***Maisha Bora***^10^												
Control	0.33 (1.84)		31.43 (6.43)		1.13 (1.67)				0.48 (0.61)			
Intervention		0.09 (0.40)		−0.02 (1.00)		−0.08 (0.26)		0.12 (0.21)		0.12 (0.10)		

Notes.

∗ *p* < 0.10.

∗∗ *p* < 0.05.

∗∗∗ p < 0.01. Standard errors in parentheses.

1 Single difference estimates, endline.

2 Intent to treat (ITT) estimates.

3 Double-difference estimates, midline and endline.

4 Double difference estimates, baseline and endline, nearest neighbor estimates.

5 Treatment on the treated (ToT) estimates.

6 Double difference, baseline and endline.

7 Intent to treat, ANCOVA regression.

8 Difference-in-differences weighted with entropy balancing method and selection of control variables with post-double-selection method via Lasso regressions, baseline and endline using machine learning methods. †q < .10; ††q < .05; †††q < .01. Q-values estimated following Benjamini and Hochberg (1995).

9 Inverse probability weighted, single difference; significant + after controlling for false discovery rate.

10 Propensity score weighted difference-in-difference regressions. n.c. = not computed or measured.

**Appendix Table 7:** Impact estimates on men's intrinsic and collective agency indicators (continuous), projects that collected data on men

	Intrinsic agency								Collective agency			
	Relative autonomy index score (range: 3 to −3)		Self-efficacy scale score (range: 8 to 40)		Number of situations in which intimate partner violence is not justified		Number of conditions met defining respect		Number of types of groups to which respondent belongs		Number of types of groups regarded as influential to which respondent belongs	
	Endline control mean	Impact	Endline control mean	Impact	Endline control mean	Impact	Endline control mean	Impact	Endline control mean	Impact	Endline control mean	Impact
**South Asia**												
***ANGeL***^1^^,^^2^												
Control	2.59 (0.90)		31.82 (4.55)		4.38 (1.18)		3.75 (0.64)		0.37 (0.64)		0.20 (0.48)	
T-N		0.05 (0.04)		0.06 (0.05)		0.03 (0.04)		−0.03 (0.04)		0.04 (0.04)		0.02 (0.04)
T-A		0.01 (0.04)		0.08* (0.04)		0.04 (0.04)		0.01 (0.04)		0.00 (0.04)		−0.02 (0.04)
T-AN		0.00 (0.04)		0.04 (0.05)		0.01 (0.04)		0.02 (0.04)		0.02 (0.03)		0.03 (0.04)
T-ANG		0.08∗ (0.04)		0.02 (0.05)		0.08∗ (0.04)		0.02 (0.04)		0.09∗∗ (0.04)		0.04 (0.04)
***AVC***^2^^,^^3^												
Control	2.54 (0.95)		34.02 (3.94)		4.38 (1.24)		3.7 (0.64)		0.2 (0.46)		0.11 (0.34)	
NGO trainings only		0.06 (0.26)		0.16 (0.61)		−0.14 (0.20)		−0.02 (0.11)		0.02 (0.06)		0.04 (0.04)
NAAFCO promotions only		0.10 (0.19)		−0.05 (0.50)		0.13 (0.14)		0.25∗ (0.09)		0.09 (0.06)		−0.05 (0.04)
Trainings + promotions		−0.12 (0.19)		0.06 (0.52)		−0.18 (0.21)		0.09 (0.09)		−0.11 (0.04)		−0.19∗ (0.03)
***FAARM***^2^												
Control	2.42 (1.03)		30.00 (3.42)		4.85 (0.45)		1.56 (1.37)		0.91 (1.09)		0.06 (0.26)	
Intervention		0.05 (0.04)		0.14∗∗∗ (0.04)		−0.01 (0.04)		0.09∗∗ (0.05)		0.02 (0.04)		0.02 (0.05)
***WINGS***^4^												
Control	0.42 (1.86)		31.76 (5.30)		4.65 (0.94)		3.41 (0.86)		0.27 (0.58)		0.20 (0.53)	
NI (DD)		NA		NA		−0.05 (0.05)		−0.05 (0.05)		0.06 (0.05)		0.04 (0.04)
NI (SD)		0.03 (0.04)		0.02 (0.04)		−0.01 (0.05)		0.00 (0.05)		0.02 (0.04)		0.03 (0.04)
**West Africa**												
***Grameen***^5^												
Control	1.33 (1.84)				4.52 (1.05)		2.42 (1.74)		1.78 (1.37)		3.98 (0.15)	
Intervention		−0.33∗∗ (0.15)		n.c.		−0.47∗∗∗ (0.15)		−0.02 (0.15)		0.91∗∗∗ (0.13)		0.86∗∗∗ (0.13)
***SELEVER***^6^												
Control	0.95 (1.91)		31.06 (5.06)		4.42 (1.24)		3.68 (0.64)		0.64 (0.81)		0.56 (0.76)	
SELEVER		0.00 (0.03)		0.00 (0.03)		−0.02 (0.02)		−0.02 (0.04)		0.06∗ (0.03)		0.07∗∗ (0.03)
***WorldVeg***^7^												
Control	0.15 (1.80)		32.70 (3.42)		3.71 (1.51)		3.30 (1.25)		2.07 (1.59)		1.89 (1.56)	
Intervention (ITT)		−0.22∗∗∗ (0.06)		−0.10 (0.10)		0.06 (0.07)		0.09 (0.08)		−0.01 (0.11)		0.03 (0.11)
Intervention (TOT)		−0.15∗∗∗ (0.05)		−0.07 (0.08)		0.04 (0.07)		−0.05 (0.07)		0.05 (0.07)		0.08 (0.07)
**East Africa**												
***JP-RWEE***^8^												
*Men in primary couple*												
Control	1.35 (1.53)				4.20 (1.48)		3.16 (1.20)		1.27 (0.91)		0.67 (0.84)	
With access to credit		0.02 (0.07)		n.c.		−0.06 (0.05)		0.07 (0.06)		0.08 (0.05)		0.11∗ (0.06)
Lost access to credit		−0.19∗∗∗ (0.05)		n.c.		−0.19∗∗∗ (0.06)		−0.04 (0.05)		−0.17∗∗∗ (0.05)		−0.12∗∗ (0.05)

Notes.

∗ *p* < 0.10.

∗∗ *p* < 0.05.

∗∗∗ p < 0.01. Standard errors in parentheses.

1 Single difference estimates, endline.

2 Intent to treat (ITT) estimates.

3 Double-difference estimates, midline and endline.

4 Double difference estimates, baseline and endline, nearest neighbor estimates.

5 Double difference, baseline and endline.

6 Intent to treat, ANCOVA regression.

7 Difference-in-differences weighted with entropy balancing method and selection of control variables with post-double-selection method via Lasso regressions, baseline and endline using machine learning methods. †q < .10; ††q < .05; †††q < .01. Q-values estimated following Benjamini and Hochberg (1995).

8 Inverse probability weighted, single difference; significant + after controlling for false discovery rate; n.c. = not computed or measured.

**Appendix Table 8:** Project impacts on women's instrumental agency indicators (continuous)

	No. of types of productive activities with input in decisions				Number of asset types (including agricultural land) solely or jointly owned			Number of types of credit sources, plus access to financial account				No. of types of activities with control over use of income				Time spent on paid and unpaid work, plus 0.5 x time spent on childcare				Number of important location types visited			
	Endline control mean		Impact		Endline control mean	Impact		Endline control mean		Impact		Endline control mean		Impact		Endline control mean		Impact		Endline control mean		Impact	
**South Asia**																							
***ANGeL***^1^^,^^2^rowhead																							
Control	3.45 (1.42)				4.86 (2.40)			0.85 (0.84)				3.53 (1.75)				10.58 (2.43)				0.35 (0.59)			
T-N			−0.01 (0.04)			−0.02 (0.05)				0.23∗∗∗ (0.03)				0.04 (0.05)				0.03 (0.05)				0.02 (0.04)	
T-A			0.05 (0.04)			−0.02 (0.05)				0.26∗∗∗ (0.04)				0.07 (0.05)				0.01 (0.05)				0.04 (0.04)	
T-AN			−0.01 (0.04)			−0.01 (0.05)				0.21∗∗∗ (0.03)				0.00 (0.05)				−0.00 (0.04)				0.05 (0.04)	
T-ANG			−0.00 (0.05)			0.05 (0.05)				0.23∗∗∗ (0.03)				0.03 (0.05)				−0.03 (0.04)				0.06 (0.04)	
***AVC***^2^^,^^3^rowhead																							
Control	4.09 (1.34)				3.84 (1.98)			0.95 (0.95)				3.84 (1.34)				10.84 (4.29)				0.58 (0.69)			
NGO trainings only			0.01 (0.18)			0.09 (0.39)				0.07 (0.08)				0.11 (0.19)				0.11 (0.58)				0.12 (0.08)	
NAAFCO promotions only			−0.13 (0.16)			0.22 (0.33)				−0.02 (0.1)				−0.06 (0.20)				0.07 (0.59)				−0.16∗ (0.07)	
Trainings + promotions			0.24∗ (0.19)			0.20 (0.39)				−0.06 (0.09)				0.29∗ (0.21)				−0.09 (0.64)				−0.14∗ (0.05)	
***FAARM***^2^rowhead																							
Control		3.46 (1.48)			3.35 (1.44)				1.39 (1.04)				3.68 (1.41)				14.50 (4.22)				0.62 (0.70)		
Intervention				0.14∗∗∗ (0.05)			0.08∗∗∗ (0.03)				0.05 (0.06)				0.13∗∗∗ (0.05)				0.01 (0.04)				0.03 (0.03)
***WINGS***^4^rowhead																							
Control		2.30 (1.43)			5.98 (2.35)				1.22 (0.87)				3.13 (1.70)				10.90 (3.31)				1.60 (0.78)		
NI (DD)				−0.01 (0.04)			0.04 (0.04)				−0.09∗∗ (0.04)				−0.01 (0.04)				−0.03 (0.05)				0.02 (0.04)
NI (SD)				0.04 (0.03)			0.07∗∗ (0.04)				0.02 (0.04)				0.04 (0.04)				−0.03 (0.04)				0.01 (0.04)
***Heifer (A-WEAI)***^2^^,^^5^rowhead																							
*Direct Beneficiary*rowhead																							
Control		1.67 (0.07)			7.04 (0.18)				4.14 (0.29)				0.82 (0.05)				10.76 (0.29)						
Full treatment				0.11 (0.07)			0.00 (0.02)				−0.16∗ (0.09)				−0.13 (0.09)				−0.20∗∗ (0.09)				
Values-based training				0.13∗ (0.07)			0.04∗∗ (0.01)				−0.08 (0.08)				0.20∗∗ (0.08)				0.07 (0.09)				
Goats				0.20∗∗∗ (0.07)			−0.01 (0.01)				−0.03 (0.08)				0.17∗∗ (0.08)				−0.07 (0.09)				
*Pay-it-forward beneficiary*rowhead																							
Control		1.62 (0.09)			6.83 (0.22)				3.06 (0.31)				0.67 (0.05)				11.26 (0.32)						
Full treatment				0.09 (0.08)			−0.00 (0.02)				0.09 (0.10)				0.40∗∗∗ (0.10)				−0.17∗ (0.10)				
Values-based training				0.09 (0.08)			0.06∗∗∗ (0.02)				0.21∗∗ (0.10)				0.32∗∗∗ (0.10)				0.00 (0.10)				
Goats				0.07 (0.08)			−0.02 (0.02)				0.12 (0.09)				0.19∗ (0.10)				−0.17∗ (0.10)				
**West Africa**																							
***Grameen***^6^rowhead																							
Control		6.19 (2.09)			0.08 (0.28)				3.56 (1.58)				6.89 (3.78)				2.33 (1.19)				2.82 (1.36)		
Intervention				0.57∗∗∗ (0.15)			−0.63∗∗∗ (0.14)				0.44∗∗∗ (0.15)				0.46∗∗∗ (0.15)				1.24∗∗∗ (0.14)				0.12 (0.15)
***SELEVER***^7^rowhead																							
Control		8.43 (6.60)			4.12 (1.89)				0.22 (0.48)				7.95 (6.39)				13.05 (5.75)				1.35 (1.02)		
SELEVER				−0.04 (0.04)			−0.01 (0.03)				0.03 (0.03)				−0.03 (0.03)				−0.02 (0.04)				0.03 (0.03)
***iDE***^6^rowhead																							
Control 1		2.16 (1.32)			4.33 (2.65)				0.52 (0.52)				3.87 (2.16)				10.04 (3.99)						
Motor pump (control group 1, DD)				−0.07 (0.05)			0.87∗∗ (0.05)				−0.04 (0.05)				0.02 (0.04)				0.02 (0.01)				n.c.
Control 2		2.25 (1.30)			4.38 (2.52)				0.52 (0.51)				4.02 (2.19)				10.01 (2.49)						
Motor pump (control group 2, DD)				−0.06 (0.06)			0.14∗∗ (0.06)				0.01 (0.08)				−0.03 (0.06)				0.00 (0.01)				n.c.
Motor pump spillover effects, DD)		2.25 (1.30)		−0.11 (0.08)	4.38 (2.52)		−0.01 (0.07)		0.52 (0.51)		0.11 (0.07)		4.02 (2.19)		−0.07 (0.06)		10.01 (2.49)		−0.00 (0.09)				n.c.
Control 1		2.72 (1.68)			4.40 (2.74)				0.48 (0.52)				3.03 (1.91)				7.97 (3.15)				1.86 (1.04)		
Motor pump (control group 1, SD)				0.07 (0.05)			0.11 (0.08)				−0.03 (0.04)				0.07∗ (0.04)				−0.05 (0.05)				0.01 (0.05)
Control 2		2.82 (1.69)			4.44 (2.66)				0.47 (0.52)				3.13 (1.92)				7.84 (3.23)				1.86 (1.11)		
Motor pump (control group 2, SD)				0.02 (0.10)			0.18 (0.16)				−0.06 (0.14)				−0.13 (0.09)				0.21∗∗ (0.10)				−0.17 (0.10)
Motor pump spillover effects, SD)		2.82 (1.69)		0.121 (0.15)	4.44 (2.66)		−0.37∗∗∗ (0.12)		0.47 (0.52)		0.09 (0.13)		3.13 (1.92)		−0.07 (0.15)		7.84 (3.23)		0.17 (0.11)		1.86 (1.11)		−0.22∗ (0.13)
***WorldVeg***^8^rowhead																							
Control		4.12 (1.75)			9.08 (2.77)				1.25 (1.27)				4.10 (2.18)				10.71 (3.10)				1.62 (1.12)		
Intervention (ITT)				−0.05 (0.10)			−0.09 (0.08)				0.01 (0.10)				0.03 (0.09)				−0.04 (0.08)				−0.02 (0.08)
Intervention (TOT)				0.08 (0.70)			−0.06 (0.06)				0.15∗ (0.08)				0.19∗∗ (0.09)				−0.07 (0.07)				0.05 (0.06)
**East Africa**																							
***JP-RWEE***^9^																							
*All women*rowhead																							
Control		3.34 (1.15)			6.10 (1.70)				0.46 (0.65)				3.70 (1.29)				9.77 (3.78)				3.68 (9.91)		
With access to credit				−0.02 (0.04)			0.10∗∗ (0.04)				−0.03 (0.05)				−0.03 (0.04)				0.02 (0.04)				0.01 (0.03)
Lost access to credit				−0.05 (0.05)			−0.02 (0.05)				−0.26∗∗∗ (0.05)				−0.05 (0.05)				0.17∗∗∗ (0.05)				0.05 (0.04)
*Women in primary couple*rowhead																							
Control		3.42 (1.10)			6.22 (1.67)				0.47 (0.67)				3.76 (1.23)				10.16 (3.69)				3.83 (10.90)		
With access to credit				−0.03 (0.05)			0.09∗ (0.05)				−0.07 (0.06)				−0.04 (0.05)				0.03 (0.05)				−0.05 (0.04)
Lost access to credit				−0.02 (0.05)			−0.00 (0.07)				−0.22∗∗∗ (0.06)				−0.02 (0.05)				0.20∗∗∗ (0.06)				0.03 (0.05)
*Maisha Bora*^10^rowhead																							
Control		3.49 (0.51)			0.66 (0.45)				0.88 (0.79)				1.57 (1.16)				18.12 (8.40)				2.05 (1.11)		
Intervention				0.10 (0.15)			−0.18 (0.11)				0.09 (0.15)				0.26∗ (0.13)				1.59 (1.75)				0.12 (0.17)

Notes.

∗ *p* < 0.10.

∗∗ *p* < 0.05.

∗∗∗ p < 0.01. Standard errors in parentheses.

1 Single difference estimates, endline.

2 Intent to treat (ITT) estimates.

3 Double-difference estimates, midline and endline.

4 Double difference estimates, baseline and endline, nearest neighbor estimates.

5 Treatment on the treated (ToT) estimates.

6 Double difference, baseline and endline.

7 Intent to treat, ANCOVA regression 9;(IDE).

8 Difference-in-differences weighted with entropy balancing method and selection of control variables with post-double-selection method via Lasso regressions, baseline and endline using machine learning methods. †q < .10; ††q < .05; †††q < .01. Q-values estimated following Benjamini and Hochberg (1995).

9 Inverse probability weighted, single difference; significant + after controlling for false discovery rate.

10 Propensity score weighted difference-in-difference regressions. n.c. = not computed or measured.

**Appendix Table 9:** Project impacts on men's instrumental agency indicators (continuous), projects that collected data on men

	No. of types of productive activities with input in decisions				Number of asset types (including agricultural land) solely or jointly owned				Number of types of credit sources, plus access to financial account				No. of types of activities with control over use of income		Time spent on paid and unpaid work, plus 0.5 x time spent on childcare		Number of important location types visited	
	Endline control mean		Impact		Endline control mean		Impact		Endline control mean		Impact		Endline control mean	Impact	Endline control mean	Impact	Endline control mean	Impact
**South Asia**																		
***ANGeL***^1^^,^^2^rowhead																		
Control	3.32 (1.29)				7.72 (1.95)				1.26 (0.97)				4.13 (1.46)		9.66 (3.30)		1.56 (0.88)	
T-N			−0.01 (0.04)				−0.02 (0.05)				0.23∗∗∗ (0.03)			0.04 (0.05)		0.03 (0.05)		0.02 (0.04)
T-A			0.05 (0.04)				−0.02 (0.05)				0.26∗∗∗ (0.04)			0.07 (0.05)		0.01 (0.05)		0.04 (0.04)
T-AN			−0.01 (0.04)				−0.01 (0.05)				0.21∗∗∗ (0.03)			0.00 (0.05)		−0.00 (0.04)		0.05 (0.04)
T-ANG			−0.00 (0.05)				0.05 (0.05)				0.23∗∗∗ (0.03)			0.03 (0.05)		−0.03 (0.04)		0.06 (0.04)
***AVC***^2^^,^^3^rowhead																		
Control		4.55 (1.16)				7.8 (2.21)				1.58 (1.06)			4.42 (1.22)		9.11 (3.37)		1.79 (0.98)	
NGO trainings only				0.16 (0.18)				0.08 (0.45)				0.12 (0.14)		0.21 (0.19)		0.35∗∗∗ (0.38)		−0.25 (0.17)
NAAFCO promotions only				0.03 (0.16)				0.16 (0.43)				−0.09 (0.12)		0.09 (0.19)		0.04 (0.32)		−0.09 (0.16)
Trainings + promotions				0.39∗∗∗ (0.16)				0.25 (0.38)				−0.12 (0.15)		0.42∗∗∗ (0.18)		0.22∗∗ (0.36)		−0.25 (0.14)
***FAARM***^2^rowhead																		
Control		4.57 (1.39)				7.55 (1.82)				1.49 (0.10)			4.37 (1.50)		10.55 (3.48)		1.88 (0.96)	
Intervention				0.09∗∗ (0.05)				0.01 (0.03)				0.14∗∗ (0.06)		0.05 (0.05)		0.01 (0.04)		0.10∗∗∗ (0.03)
***WINGS***^4^rowhead																		
Control		2.66 (1.36)				6.97 (2.01)				1.20 (0.94)			3.57 (1.48)		7.83 (3.47)		2.06 (0.77)	
NI (DD)				−0.03 (0.04)				−0.05 (0.05)				−0.02 (0.04)		−0.03 (0.04)		0.03 (0.04)		−0.05 (0.04)
NI (SD)				−0.01 (0.03)				−0.03 (0.04)				0.04 (0.04)		−0.02 (0.04)		−0.01 (0.04)		0.00 (0.04)
**West Africa**																		
***Grameen***^5^rowhead																		
Control		8.25 (1.56)				0.19 (0.39)				4.36 (0.91)			4.68 (3.74)		2.27 (1.32)		1.85 (1.38)	
Intervention				0.03 (0.14)				0.28∗ (0.15)				0.19 (0.15)		0.26∗ (0.15)		0.81∗∗∗ (0.14)		0.09 (0.15)
***SELEVER***^6^rowhead																		
Control		16.44 (7.17)				7.70 (2.10)				0.43 (0.64)			16.08 (6.84)		7.75 (4.34)		1.80 (1.13)	
SELEVER				−0.04 (0.03)				−0.00 (0.05)				0.06 (0.05)		−0.03 (0.03)		0.00 (0.05)		0.02 (0.03)
***WorldVeg***^7^rowhead																		
Control		4.71 (1.13)				10.21 (1.73)				1.63 (1.36)			5.18 (1.43)		7.25 (3.10)		2.25 (1.10)	
Intervention (ITT)				0.02 (0.09)				−0.10∗ (0.05)				−0.00 (0.05)		0.03 (0.06)		0.10 (0.08)		−0.16∗ (0.09)
Intervention (TOT)				0.05 (0.06)				−0.14∗∗ (0.06)				−0.07 (0.09)		−0.04 (0.09)		0.01 (0.09)		−0.03 (0.10)
**East Africa**																		
***JP-RWEE***^8^rowhead																		
Control		3.43 (1.10)				6.47 (1.57)				0.70 (0.71)			3.85 (1.17)		7.34 (2.89)		15.37 (42.18)	
*Men in primary couple*rowhead																		
With access to credit				−0.06 (0.05)				0.04 (0.06)				−0.10 (0.07)		−0.07 (0.05)		0.02 (0.05)		−0.03 (0.04)
Lost access to credit				0.00 (0.05)				−0.02 (0.06)				−0.21∗∗∗ (0.06)		−0.02 (0.05)		0.20∗∗∗ (0.06)		0.02 (0.05)

Notes.

∗ *p* < 0.10.

∗∗ *p* < 0.05.

∗∗∗ p < 0.01. Standard errors in parentheses.

1 Single difference estimates, endline.

2 Intent to treat (ITT) estimates.

3 Double-difference estimates, midline and endline.

4 Double difference estimates, baseline and endline, nearest neighbor estimates.

5 Double difference, baseline and endline.

6 Intent to treat, ANCOVA regression;^9^(IDE).

7 Difference-in-differences weighted with entropy balancing method and selection of control variables with post-double-selection method via Lasso regressions, baseline and endline using machine learning methods. †q < .10; ††q < .05; †††q < .01. Q-values estimated following Benjamini and Hochberg (1995).

8 Inverse probability weighted, single difference; significant + after controlling for false discovery rate. n.c. = not computed or measured.

**Appendix Table 10:** Project impacts on women's and men's empowerment (whether empowered and empowerment score) and gender parity

	Women				Men				Whether household achieved gender parity	
	Whether empowered		Empowerment score		Whether empowered		Empowerment score			
	Endline control mean (%)	Impact	Endline control mean	Impact	Endline control mean (%)	Impact	Endline control mean	Impact	Endline control mean (%)	Impact
**South Asia**										
***ANGeL***^1^, ^2^										
Control mean	0.25		0.59		0.39		0.67		0.47	
T-N		0.08∗∗		0.04∗∗∗		0.10∗∗		0.03∗∗		0.05
T-A		0.07∗		0.04∗∗		0.02		0.01		0.05
T-AN		0.08∗∗		0.04∗∗∗		−0.01		−0.00		0.08∗
T-ANG		0.13∗∗∗		0.07∗∗∗		0.01		0.00		0.13∗∗∗
***AVC***^2^^,^^3^										
Control mean	0.23		0.53		0.41		0.18		0.31	
NGO trainings only		0.01		0.02		0.02		−0.00		0.12∗
NAAFCO promotions only		−0.03		0.02		0.08		0.02		−0.05
Trainings + promotions		−0.09∗		0.01		−0.02		−0.02		0.02
***FAARM***^2^^,^^4^										
Control mean	0.04		0.47		0.20		0.61		0.28	
Intervention	0.24	2.1∗∗∗	0.61	0.13∗∗∗	0.25	0.30	0.63	0.02∗∗	0.54	1.31∗∗∗
***WINGS***										
A-WEAI 5										
Control (Standard)	0.13		0.79		0.29		0.90		0.507	
Nutrition-intensification		−0.22		0.01		−0.05		−0.02		−0.01
Pro-WEAI 3										
Control (Standard)	0.57		0.65		0.72		0.79		0.70	
Nutrition-intensification		0.02		−0.02		−0.04		−0.02		0.02
***HEIFER***^2^^,^^6^										
Heifer Direct beneficiaries										
Control	0.54		0.75							
Full treatment		0.10∗		0.06∗∗∗		n.c.		n.c.		n.c.
Values-based training		0.06		0.05∗∗		n.c.		n.c.		n.c.
Goats		0.08		0.05∗∗		n.c.		n.c.		n.c.
Heifer Pay-it-forward beneficiaries										
Control	0.48		0.71							
Full treatment		0.07		0.07∗∗∗		n.c.		n.c.		n.c.
Values-based training		0.13∗∗		0.08∗∗∗		n.c.		n.c.		n.c.
Goats		0.07		0.03		n.c.		n.c.		n.c.
**West Africa**										
***Grameen***^7^										
Intervention		−0.08		0.00		0.15∗∗		0.06∗∗∗		−0.06
***SELEVER***^8^										
Control	0.14		0.53		0.43		0.68		0.29	
SELEVER		0.03		0.00		0.03		0.01		0.02
SELEVER+		−0.02		−0.02		0.04		0.01		−0.06
***iDE***^7^										
Control 1 (A-WEAI			0.75							
Motor pump				−0.00		n.c.			n.c.	
Control 2 (A-WEAI)			0.75							
Motor pump				−0.02		n.c.		n.c.		n.c.
Spillover control (A-WEAI)			0.75							
Spillover effect				−0.07∗		n.c.		n.c.		n.c.
Control 1 (Pro-WEAI			0.69							
Motor pump				0.02		n.c.		n.c.		n.c.
Control 2 (Pro-WEAI)			0.69							
Motor pump				−0.03		n.c.		n.c.		n.c.
Spillover control (Pro-WEAI)			0.69							
Spillover effect				−0.05		n.c.		n.c.		n.c.
***WorldVeg***^9^										
Control	0.29		0.65		0.70		0.89		0.36	
Intervention ITT^2^		0.19∗∗∗†		0.04		−0.25∗∗∗††		−0.12∗∗∗††		0.25∗∗∗††
Intervention ToT^6^		0.16∗∗		0.08∗∗		−0.25∗∗		−0.09∗∗		0.24∗∗∗†††
**East Africa**										
***JP-RWEE***^10^										
Control										
With access to credit		0.03		0.03		0.07		0.05∗∗		−0.00
Lost access to credit		−0.46∗∗+		−0.12∗∗+		−0.47∗∗+		−0.12∗∗+		−0.39∗∗+
***Maisha Bora***^11^										
Control	0.21		0.54		0.38		0.64		0.39	
Intervention		0.10		0.06		0.12		0.02		−0.05

Notes.

∗ *p* < 0.10.

∗∗ *p* < 0.05.

∗∗∗ p < 0.01.

1 Single difference estimates, endline.

2 Intent to treat (ITT) estimates.

3 Double-difference estimates, midline and endline.

4 Multilevel logistic regression with random effects, OR = odds ratio.

5 Double difference estimates, baseline and endline, nearest neighbor estimates.

6 Treatment on the treated (ToT) estimates.

7 Double difference, baseline and endline.

8 Intent to treat, ANCOVA regression.

9 Difference-in-differences weighted with entropy balancing method and selection of control variables with post-double-selection method via Lasso regressions, baseline and endline using machine learning methods. †q < .10; ††q < .05; †††q < .01. Q-values estimated following Benjamini and Hochberg (1995).

10 nverse probability weighted, single difference; significant + after controlling for false discovery rate.

11 Propensity score weighted difference-in-difference regressions. n.c. = not computed or measured.

## Data Availability

The data used in the paper are in the Appendix Tables

## Footnotes

9 Some of the projects that had started earlier used A-WEAI in their baselines, but all, except for Heifer, use pro-WEAI in their endline surveys. A-WEAI (Abbreviated WEAI) (Malapit et al., 2017) has the five domains of WEAI, but only six indicators, and was developed to reduce survey length and to eliminate questions that were difficult to implement in the field. The corresponding cut-off for A-WEAI is 80%: an individual is defined as empowerment if she has an empowerment score of at least 80%. A-WEAI can be computed from surveys collecting pro-WEAI. See Appendix Table 5 for comparisons between A-WEAI and pro-WEAI.

## Footnotes

2 We use the 12-indicator version of pro-WEAI, as presented in Malapit et al., 2019. Pro-WEAI has been subsequently revised to 10 indicators, as discussed in Seymour et al. (2023). The indicator for respect among household members was dropped because the indicator could not be calculated in households with only a single adult, which resulted in these households being dropped from the sample. Membership in influential groups was dropped because it was closely correlated with group membership, and hence, did not provide additional information on collective agency.

3 We mapped strategies to types of agency ex post, as the projects had been designed (and funded) when GAAP2 started.

4 The TRAIN endline was halted because of COVID-19; eventually a phone survey was fielded to track some of the project's outcomes. The endline survey was eventually fielded and completed between January to March 2022. MoreMilk restarted the intervention in a different community so no endline data were collected.

5 Working groups for each topic area created a matrix listing the detailed questions and methods of data collection. The research team honed this matrix to determine which questions were most essential. The pro-WEAI qualitative guidance include seven protocols (Appendix Table 3): (1) review of project documents; (2) community profile; (3) seasonality calendar; (4) local understandings of empowerment; (5) life histories; (6) key informant interviews with market traders; and (7) key informant interviews with project staff.

6 Some of the projects had already implemented baseline surveys using an earlier WEAI version, the Abbreviated WEAI (A-WEAI) (Malapit et al., 2017) when they joined GAAP2. In separate work, Seymour et al. (2023) find that differences in the impact estimates on the aggregate metrics depending on the WEAI variant used are small in magnitude but may be significant. We take the variation in empowerment indicators into account in analyzing the results across the portfolio. Some projects did not collect all 12 pro-WEAI indicators, because some (such as self-efficacy) were considered optional when the baselines were implemented, and a few projects did not collect data on men.

7 Efforts to streamline the pro-WEAI questionnaire are ongoing. The number of questions in the 2019 version of pro-WEAI ranged from 69 to 154, depending on skip patterns. Following further validation, this was reduced to a range of 54–126 in the 2022 version (Seymour et al., 2023). We are developing a shorter version using optimization techniques; the indicative range is between 39 and 65 questions.

8 Saturation is a theoretical concept in qualitative methods, in which no new themes, ideas, or patterns continue to emerge from the data despite gathering more data from additional respondents.

10 Heifer collected only the six A-WEAI indicators, but we include Heifer in the analysis to increase geographic representation.

11 This is estimated using “stdbeta, se store” in Stata Version 17.0.

12 The thresholds vary for each indicator because the indicators capture different aspects of agency and may be measured in different units. For example, a person is defined as adequate with respect to asset ownership if she or he owns, either solely or jointly, at least ONE of the following: 1) At least THREE small assets (poultry, nonmechanized equipment, or small consumer durables); 2) At least TWO large assets; 3) Land. In contrast, the adequacy with respect to work balance is achieved if the person works less than 10.5 h per day, where workload = time spent in primary activity + (1/2) time spent in childcare as a secondary activity.

13 The five circumstances are: (1) going out without telling him; (2) neglecting the children; (3) arguing with him; (4) refusing to have sex with him; and (5) burning the food. These were adapted from questions in the Demographic and Health Surveys.

14 A subset of beneficiaries in JP RWEE lost access to credit either because they did not repay their loan or because they left the savings and credit group (Hillesland et al., 2022).

15 The negative estimated impact on the number of types of credit decisions made was only detected in the double-difference estimates. The estimated impact was not significantly different from zero using single-difference estimates.

16 While adding questions on intrinsic agency (e.g., questions on confidence) to the quantitative questionnaire could ensure pro-WEAI measures the right constructs, doing so is only an option if a round of exploratory qualitative work is completed prior to the quantitative baseline and the qualitative findings reveal unanticipated pathways to program impact.

17 When baseline data for the GAAP2 projects was collected, pro-WEAI was still in development. The earlier A-WEAI can be calculated from pro-WEAI and could have been used to diagnose some areas of disempowerment from among the A-WEAI indicators (see Appendix Table 5 for a comparison of pro-WEAI and A-WEAI). However, the projects themselves had already been approved prior to their joining GAAP2. None of them, except for ANGeL, used WEAI findings to diagnose empowerment gaps and to design the intervention. Now, that pro-WEAI is established and collected more broadly, projects can diagnose areas of disempowerment from their own baseline data or based on data collected from projects operating in similar areas.
